# A Sensitive Thermoelectric Respiratory Sensor Using a Hollow‐Square Structure of Cubic Silicon Carbide‐Based Heterojunction

**DOI:** 10.1002/smll.202510634

**Published:** 2026-01-20

**Authors:** Thi Lap Tran, Duy Van Nguyen, The Lai Khanh, Thien Hoang, Toan Trong Tran, Pingan Song, Nam‐Trung Nguyen, Dzung Viet Dao, John Bell, Ravinesh C. Deo, Toan Dinh

**Affiliations:** ^1^ School of Engineering University of Southern Queensland Springfield Central Queensland Australia; ^2^ School of Electrical and Data Engineering University of Technology Sydney Ultimo New South Wales Australia; ^3^ Queensland Quantum and Advanced Technologies Research Institute Griffith University Brisbane Queensland Australia; ^4^ School of Engineering and Built Environment Griffith University Gold Coast Queensland Australia; ^5^ Centre for Future Materials University of Southern Queensland Springfield Central Queensland Australia; ^6^ School of Mathematics, Physics and Computing University of Southern Queensland Springfield Central Queensland Australia

**Keywords:** 3C‐SiC, heterojunction, hollow‐square structure, temperature gradient, thermal resistance

## Abstract

Respiratory rates play a crucial role in health assessment and rehabilitation. However, current respiratory monitoring devices often rely on metal‐ or polymer‐based sensors, and skin‐mounted electronics, face challenges such as low sensitivity, limited durability/stability, and substantial power demands in complex respiratory environments. Herein, we introduce innovations in design and fabrication of a hollow‐square cubic silicon carbide (3C‐SiC)‐based self‐powered sensor, which operates via the Seebeck effect in 3C‐SiC/Si heterojunction, for respiratory rate monitoring. Through manipulating thermal transport, this design significantly enhances airflow sensing performance, yielding a thermal voltage output approximately 3.5 times higher than that of conventional solid structures. The sensor exhibits remarkable repeatability and durability, maintaining stable voltage responses across 1000 airflow testing cycles. Moreover, elevated temperatures drive a transition from conventional Seebeck effect in a single semiconductor layer to heterojunction‐driven effects, resulting in a higher thermal voltage and enabling further sensor optimization under high‐temperature environments. By integrating this hollow‐square 3C‐SiC sensor into a functional mask, the sensor enables real‐time monitoring respiratory rate of workers in hot environments. An integrated alarm system provides alerts to the users in response to sudden changes in their respiratory rates, offering a reliable and practical tool for continuous health surveillance of workers in high‐temperature environments.

## Graphical abstract

A sensitive self‐powered respiratory sensor is developed using a hollow‐window structure implemented on a 3C‐SiC/Si heterojunction‐based platform. This design enables the manipulation of thermal transport with the heterojunction regulating the flow of thermally generated charge carriers, yielding a significantly higher output thermal voltage than that of conventional designs.

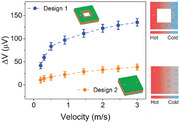

## 1 Introduction

Hot working environment is a leading cause of heat‐related illness among outdoor laborers such as roofers, road construction crews, miners, and agricultural workers, who face prolonged exposure to high‐temperature conditions [1, 2]. In the US, approximately ten million workers are working in such harsh environments annually [3], contributing to over 120 000 incident‐related fatalities and an estimated $190 billion in healthcare costs each year [4]. Monitoring vital signs of outdoor workers in these high‐temperature working settings is crucial, as it provides early warning signs of their abnormal health conditions, helps prevent incidents, and improves overall productivity [5, 6]. Among vital signs, such as body temperature, respiratory rate, and heart rate, a sudden change in respiratory rate is a readily observable sign of illness [6, 7, 8]. Respiratory rate is defined as the number of breaths a person takes per minute.

Traditional monitoring of respiratory rate is unsuitable for hot, outdoor settings. In the clinical setting, respiratory rate is predominantly screened through manual counting by clinicians, which can lead to inaccuracies and challenges in continuous long‐term monitoring [9]. Alternatively, respiratory rate can be measured using various methods, including spirometry, capnometry, and pneumography [10, 11]. However, these techniques are complex, can cause discomfort for patients, and require trained staff to operate, making them impractical for real‐time on‐site monitoring. Therefore, there is a pressing need to develop wearable sensors for accurate and continuous respiratory rate monitoring.

Wearable thermal flow respiration sensors have attracted significant interest due to their potential for monitoring respiratory rate in daily life [12, 13, 14, 15, 16]. However, most of these devices rely on external power sources, which pose challenges for long‐term monitoring and raise environmental concerns. Recent advances in respiratory monitoring have shifted toward self‐powered thermoelectric sensors, which overcome these limitations by converting the temperature difference between exhaled air and the surrounding environment into a measurable thermal voltage following the popular equation *V*  =  *S* × Δ*T* with S being the Seebeck coefficient and Δ*T* being a temperature gradient [17, 18, 19, 20]. As a result, it is demanding to take advantage of materials with a high Seebeck coefficient or devise structures to boost the temperature gradient.

Despite significant advancements in thermoelectric materials and sensor design, thermoelectric devices still face challenges in practical applications due to their low output performance and material degradation [21, 22, 23, 24, 25]. For instance, a wearable respiration sensor by Yu et al. [21] used an ultrathin vertical structure of Bi_2_Te_3_ and Sb_2_Te_3_, achieving a fast response time but producing a low voltage. This was attributed to challenges in designing an effective heat absorber and heat sink. Furthermore, Bi_2_Te_3_ has poor mechanical stability and is toxic, which raises major concerns for direct contact with the skin [26]. Similarly, Wu et al. [24] developed a temperature sensor featuring a sandwich‐like structure composed of MWCNTs and MXene nanosheets. This architecture forms a ternary heterojunction that optimizes charge carrier transport pathways, improving the Seebeck coefficient. However, MXene suffers from poor thermal oxidative stability and gradually oxidizes when exposed to air [27], posing challenges for long term monitoring. Moreover, He et al. [25] developed a self‐powered respiratory monitoring sensor based on thermoelectric fabrics (CNTs/PEDOT:PSS), fabricated by applying the thermoelectric fabrics onto cotton via a layer‐by‐layer self‐assembly strategy. Although this design enhances the fabric's electrical conductivity and durability, the overall output performance remains low due to the relatively small Seebeck coefficient. Besides, conventional thermal sensors with a solid structure further restrict sensing performance due to low temperature gradient [28]. To achieve high‐performance and long‐term monitoring, it is crucial to use materials with a high Seebeck coefficient in combination with innovative designs. The design must facilitate a large temperature gradient between the hot and cold terminals to generate a high thermal voltage during respiration [23, 26, 29]. Additionally, the selected materials should not only have a high Seebeck coefficient but also possess stable material integrity and resistance to liquids and chemicals, which are essential for generating significant voltage changes and ensuring long‐term sensor operation [20, 23]. Cubic silicon carbide (3C‐SiC) has been known as a superior material due to its excellent thermal properties [30, 31] and outstanding chemical inertness, which hardly reacts the local chemicals in most corrosive environments [32, 33]. It is also known for its biocompatibility [34, 35], making it safe for contact with the human body. Additionally, 3C‐SiC is the only SiC polytype that can be epitaxially grown on large, low‐cost Si wafers [36, 37], enabling the mass production of wearable sensors. Finally, SiC forms a heterojunction to enhance charge separation and performance of thermoelectric sensors, it works as a coating/protecting layer to humidity and other environmental conditions.

In this work, we introduce a thermoelectric respiratory sensor that uses a hollow‐square structure of 3C‐SiC/Si heterojunction for respiratory monitoring. The unique design of a square hollow structure implemented on a semiconductor heterojunction‐based platform, which enables manipulation of thermal transport, while the heterojunction regulates the transport of thermally generated charge carriers. This design maximizes the material thermal resistance, disrupting the continuous heat transport pathway across the device, thereby improving the output thermal voltage compared to the solid structure. Under a controlled temperature condition, the hollow structure exhibits thermal voltage variations approximately 3.5 times greater than those of the conventional solid design under identical testing conditions. Increasing the heat source temperature or airflow velocity further improves the flow sensor's performance. The 3C‐SiC/Si exhibits a relatively high Seebeck coefficient, ranging from approximately 77 to 102 µV K^−1^ within the temperature range from 303 to 343 K. The sensor also demonstrates negligible changes in the voltage response over 1000 air flow cycles, indicating its excellent stability. Furthermore, there is a transition from the conventional Seebeck effect of n‐type semiconductor to large voltage responses under the effect of heterojunction at higher temperatures (e.g. greater than 343 K), providing a foundation for further optimization of 3C‐SiC/Si heterojunction‐based device for applications at high‐temperature environments, such as deep‐mine or firefighting conditions. For demonstration, the 3C‐SiC hollow‐square sensor can successfully monitor different human respiratory rates in hot environments, making it suitable for health monitoring of workers in outdoor settings. We also developed a smart alarm system that alerts users when their breathing rate suddenly rises over 25 breaths per minute, or when breathing ceases for more than 10 s. These promising results could advance future research on developing high‐performance self‐powered wearable respiratory sensors for monitoring physical health and providing timely alerts.

## 2 Result and Discussion

### 2.1 Structural Designs of Thermoelectric 3C‐SiC/Si Sensors

**FIGURE 1:**
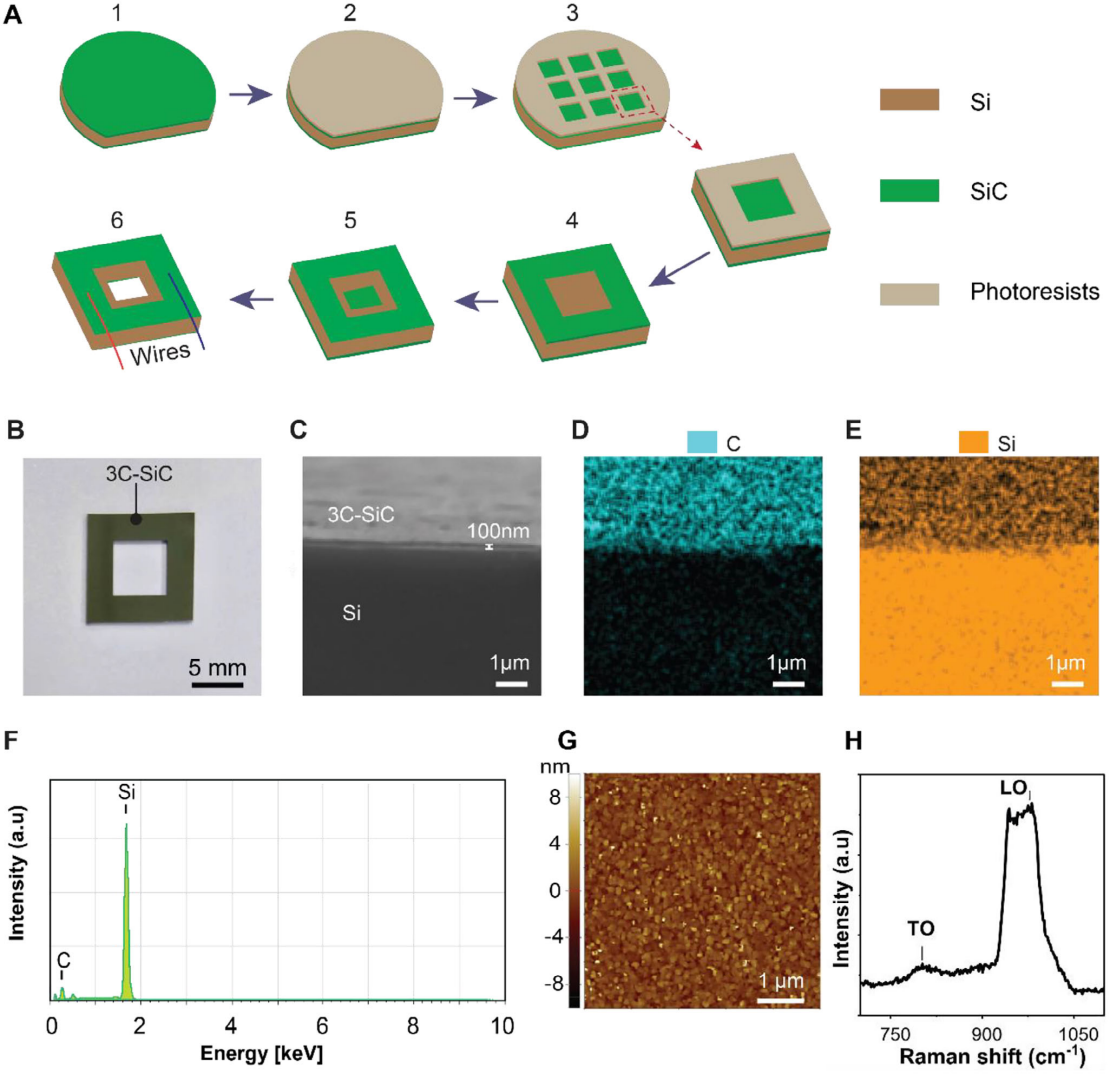
Fabrication process and material characterization. (A) The fabrication steps. (1) Deposition of 3C‐SiC thin film on both sides of a Si wafer; (2) Spin‐coating of photoresist on one side of the SiC; (3) Patterning the photoresist; (4) Dry etching of the SiC layer to open square windows; (5) Wet etching of the Si substrate to form the hollow square; and (6) Remove the SiC membrane to form a hollow‐window structure. (B) Optical image of the hollow‐window structure device. (C–E) SEMimage of the 3C‐SiC/Si heterojunction and the corresponding EDS elemental mapping for C and Si in 3C‐SiC/Si. (F) EDS spectrum of C and Si in 3C‐SiC/Si. (G) AFM image of the 3C‐SiC thin film, showing surface morphology. (H) Raman spectra of the 3C‐SiC/Si sample.

We designed a 3C‐SiC/Si hollow‐square structure to enable an increase in the device's thermal resistance and create a steeper temperature gradient across the sensor. This design aims to enhance output performance by increasing the temperature gradient across the device. Figure 1A illustrates the fabrication process of the thermoelectric sensor based on 3C‐SiC/Si. The process began with the growth of 100 nm thick cubic silicon carbide (3C‐SiC) thin films on both sides of a Si wafer using low‐pressure chemical vapor deposition (LPCVD) at 1000°C (step 1). To enable high conductivity of SiC, we utilized nitrogen doping with a carrier concentration of approximately 10^19^ cm^−3^. The surface of the 3C‐SiC was then spin‐coated with a 1.5 µm‐thick layer of AZ5214 photoresist, followed by soft‐baking at 100°C for 1 min (step 2). The photoresist was patterned using ultraviolet exposure via photolithography (step 3). Dry‐etching of the SiC layer was carried out using inductive coupled plasma to open square windows (step 4). The Si layer was then wet etched in 30 wt.% KOH solution at 80°C for approximately 10 h until the hollow window was opened (step 5). Finally, the SiC membrane layer was removed to form the hollow‐square 3C‐SiC/Si structure (step 6). The sample was cleaned with acetone and isopropanol and then dried out with nitrogen gas to remove any remaining contaminants before making an electrical interconnection.

Figure 1B shows an optical image of the fabricated device, featuring an outer square with 10 mm side length and a hollow square with a 5 mm side length. Here, 3C‐SiC acts as the sensing material, with the hollow‐square window design to enhance the temperature gradient between the two electrode regions of the device when an elevated temperature is applied at one end. Figure 1C displays the Scanning Electron Microscopy (SEM) image of the 3C‐SiC/Si, confirming that an approximately 100‐nm thick 3C‐SiC layer was successfully grown on the Si substrate (Figure S1). Figure 1D–F presents the corresponding Energy Dispersive Spectroscopy (EDS) elemental mappings for C and Si, along with the EDS spectrum, highlighting the distribution of C and Si. It is clearly seen that the Si substrate (bottom) contains only Si, while SiC layer (top) contains both Si and C. Furthermore, Figure 1G displays the atomic force microscopy (AFM) image of the 3C‐SiC thin film, acquired over a 5 × 5 µm^2^ area. The root means square roughness was measured to be 1.49 nm, indicating the even growth of the 3C‐SiC layer. Finally, the Raman spectrum of the 3C‐SiC/Si was measured in the range of 700–1150 cm^−1^ (Figure 1H), confirming the formation of the thin 3C‐SiC layer on the silicon substrate. The material exhibits a transverse optical (TO) peak at 797 cm^−^^1^ and a longitudinal optical (LO) peak at 975 cm^−^^1^, consistent with previously reported results [38, 39]. The Raman spectrum of 3C‐SiC/Si in the range of 400–1150cm^−1^ was also investigated (Figure S2), indicating crystalline Si peak (LO phonon mode) around 519 cm^−1^. These results further confirm the successful growth of 3C‐SiC layer on Si substrate.

### 2.2 Thermal Sensing Performance of a Hollow‐Square 3C‐SiC/Si Sensor

**FIGURE 2:**
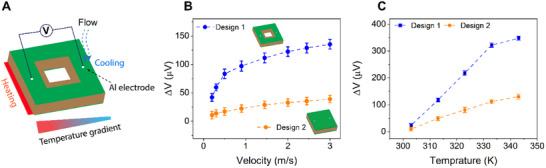
Comparison of the voltage variation of the 3C‐SiC/Si sensors with two different designs. (A) Schematic diagram of the hollow‐square 3C‐SiC/Si sensor under test. (B) Voltage variations of the two designs under various airflow velocities at a heat source of 313 K. (C). Voltage variations of the two designs under various temperatures at a flow velocity of 2.45 m/s.

To investigate the performance of the 3C‐SiC/Si sensor, we constructed a custom‐built testing platform. As shown in Figure 2A, the device was mounted on a Linkam temperature controller (MFS350), which served as the heat source. The temperature of the heat source was precisely controlled using the LINK software connected to the MFS350. Airflow was generated using a Jun‐Air compressor connected to a pressure controller (ElveFlow MK3+), function as the cooling source. Additional experimental details are provided in the Experimental section. In our experiment, the temperature controller acted as the heat source, and the airflow served as the cooling source that increased the temperature gradient between the two SiC electrode areas, resulting in an output voltage. To demonstrate the effectiveness of the hollow‐square structure (Design 1), the 3C‐SiC/Si conventional solid structure with the same dimensions (Design 2) was also fabricated. More details about the dimensions of the devices are illustrated in Figure S3. Both designs were tested under identical heating and airflow conditions. Heat was applied on one side to simulate hot environments, while the cooling flow was applied on the other side to simulate human breathing flow. The presence of a hollow in Design 1 increases its thermal resistance and therefore maintains a larger temperature gradient between the hot and cold ends compared to that of Design 2. Therefore, the voltage variation in Design 1 is higher than that in Design 2.

Figure 2B displays the voltage variations (Δ*V*)—defined as the thermal voltage difference with and without airflow—of both designs with respect to different airflow velocities at a heat source temperature of 313 K. When airflow is applied to cool the cold SiC electrode area, its temperature decreases, which increases the temperature gradient between the hot and cold regions. As a result, the thermal voltage rises. Increasing airflow velocity strengthens the cooling effect [34, 40], leading to a larger temperature gradient as well as higher thermal voltage. In comparison, Design 1 produces voltage changes approximately 3.5 times higher than those of Design 2.

Moreover, the sensing responses to airflow is controlled by heating temperature on the hot side (Figure 2C). The electrical signals were recorded at a flow velocity of 2.45 m/s applied to the cold side, while the temperature of the heat source on the hot side varied from 303 to 343 K. The results indicate that thermal voltage changes of Design 1 improve approximately threefold than those of Design 2. One factor contributes to such excellent results is the high Seebeck coefficient of 3C‐SiC/Si material (Figure S4), which ranges from 77 to 102 µV K^−1^ within the temperature controller from 303 to 343 K at the hot electrode. This value is comparable to that of n‐type heavy‐doped polycrystalline SiC [41], and β‐SiC nanowires [42]. Furthermore, this value is significantly higher than that of several state‐of‐the‐art flexible thermoelectric materials for wearable sensing, such as PEDOT:PSS/SF (23 µV K^−1^) [43], and MXene/CNT/PEDOT:PSS (9.8 µV K^−1^) [17]. The detailed comparison of the Seebeck coefficient from this work with other materials is presented in Table S1. On the other hand, the thermal voltage variations are observed to saturate at high temperatures. This can be attributed to the combination effect of the heterojunction, which drives thermal generated electrons from Si to SiC, and the reducing electron mobility in the 3C‐SiC at high temperature [34, 44]. A detailed explanation is discussed later in the manuscript.

Furthermore, we consider the charge carriers movement within 3C‐SiC under low temperatures (less than 343 K) for the targeted sensor application in general hot working environments. Since the two electrodes are deposited on 3C‐SiC layer, charge carriers in this material participate in sensing mechanisms. First, when n‐type 3C‐SiC (with a bandgap of 2.38 eV) and p‐type Si (with a bandgap of 1.12 eV) [30, 34] are brought together, a heterojunction is formed. Here, electrons diffuse from the 3C‐SiC side to the Si side, while holes migrate from the Si to the 3C‐SiC. The movement of these charges creates a depletion region, and a built‐in electric field pointing from 3C‐SiC to Si [44, 45]. At equilibrium, this field stops further the net charge movement, and the Fermi levels of both materials become aligned (Figure S5). Second, when the 3C‐SiC/Si is heated, thermal energy excites charge carriers in both 3C‐SiC and Si layers [44, 46]. Once this energy surpasses the activation energy of impurities, charge carriers are fully generated [44]. In the 3C‐SiC layer, the donors are excited by thermal energy, contributing electrons as charge carriers in its conduction band. Simultaneously, the minority charge carrier (electrons) are generated in the Si substrate and pumped to the SiC layer thanks to the built‐in electric field in the heterojunction [34]. At a temperature below 343 K as in this work, the contribution of the minority charge carriers in p‐type Si to the n‐type 3C‐SiC layer is very small compared to that of the impurities in the SiC [31, 44]. Therefore, the donor‐generated electrons in the n‐type 3C‐SiC are considered to be the main charge carriers under these low temperatures, and the movement of electrons in 3C‐SiC follows the Seebeck effect in n‐type semiconductors. However, the charge carrier behavior can be different under higher temperatures, which is discussed in more detail in the latter section.

**FIGURE 3:**
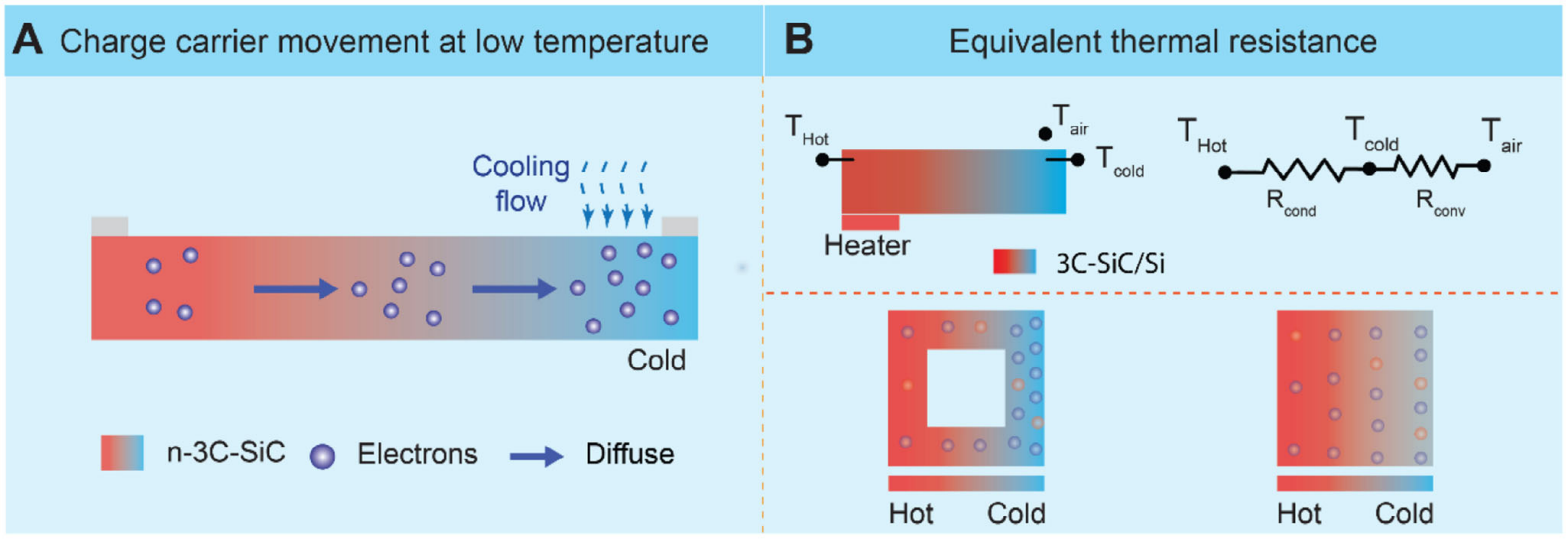
Working mechanism of the 3C‐SiC/Si heterojunction thermoelectric sensor at low heating temperature. (A) Electron diffusion in n‐3C‐SiC layer by asymmetric heating/cooling (applied temperature lower than 343 K). (B) Equivalent thermal circuit for both Designs (top) and their equivalent electron movements (bottom).

Under low temperature (less than 343 K), electrons on the hot side of 3C‐SiC gain more energy and move faster than those on the cold side (Figure 3A). As a result, electrons diffuse from the hot side to the cold side, generating a potential difference between the two ends [23, 44]. This results in the generation of thermoelectric voltage *V* between the two electrodes. The thermal voltage between two electrodes is described as follows [44, 47],

(1) V=S×ΔT

where *S* represents the Seebeck coefficient of the material, and Δ*T*  = *T*_hot_  − *T*_cold_ is the difference in temperature between the hot end (*T*_hot_) and the cold end (*T*_cold_). The output thermal voltage V of the device is proportional to the temperature difference (*∆T*). Therefore, a window‐etched structure (Design 1) increases its thermal resistance, which enhances the temperature difference between the two electrodes, thereby contributing to an improvement in thermal output voltage [23, 26, 28, 48].

Since the 3C‐SiC layer is hundreds of times thinner than the Si substrate, the thermal resistance and heat capacity of the 3C‐SiC/Si are similar to those of Si [49]. Assuming that the convection at the hot side is negligible compared to the cold side, the simplified equivalent thermal circuits for both Designs and their equivalent electron movements are shown in Figure 3B (top) and (bottom), respectively. The temperature of the cold end can be expressed as:

(2) $$T_{\mathrm{cold}}=T_{\mathrm{hot}}\;-\frac{\left(T_{\mathrm{hot}}-T_{\mathrm{air}}\right)\;R_{\mathrm{Cond}}}{R_{\mathrm{Cond}}+R_{\mathrm{Conv}}}$$

where $R_{\mathrm{cond}}=\frac{L}{k.A}$ is the thermal conduction resistance of the 3C‐SiC/S structure, determined by the material's thermal conductivity (*k*),  cross‐sectional area (*A*), and thickness L. While $R_{\mathrm{conv}\;}=\frac{1}{h.A_{s}}$ is the external convection resistance introduced by cooling airflow, where *h* is the convection heat transfer coefficient and *A*_s_ is the effective surface area exposed to the airflow. *A*_s_ is considered the same for both designs due to our experimental setup. Therefore, when the temperature on the hot side and airflow velocity are held, *T*_air_, *T*_hot_, *R*_Conv_ in Equation (2) are considered unchanged for both Designs. According to Equation (2), an increase in *R*_Cond_ results in a decrease in *T*_cold_, thereby enhancing the thermally induced output voltage of Design 1 compared to Design 2. Furthermore, raising airflow velocity leads to an increase in different temperatures between the two ends [40].

The hollow‐ etched of Design 1 increases its *R*_Cond_, leading to a lower cold‐side temperature compared with Design 2. The corresponding temperature gradient across both designs is shown in Figure 3B (bottom). As a result, Design 1 exhibits a larger temperature difference between its hot and cold ends, which contributes to an improved thermal output voltage relative to Design 2. The example of the real‐time gradient temperature response between the hot and cold electrodes in Design 1 and Design 2 is shown in Figure S6.

**FIGURE 4:**
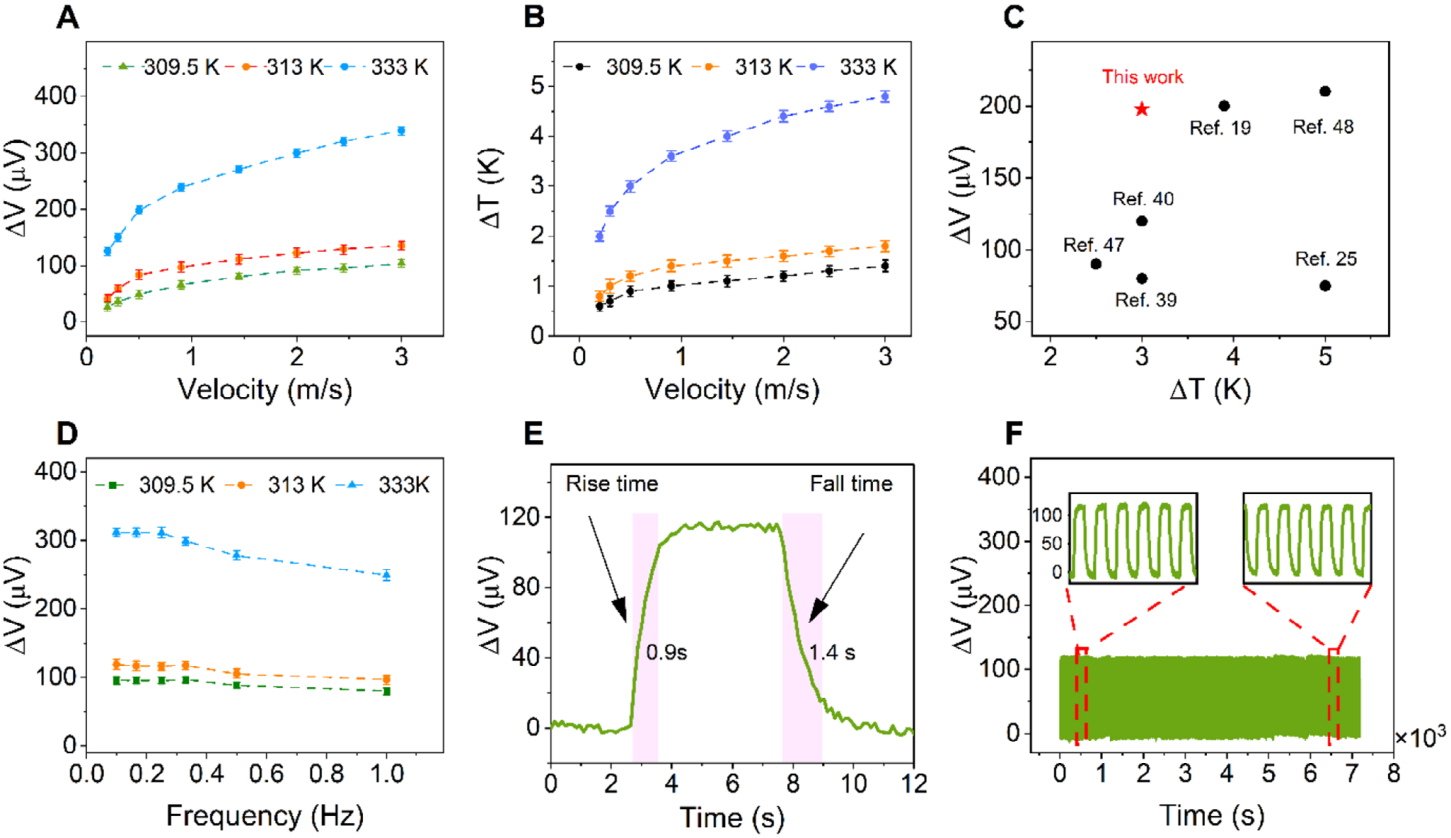
Performance of the hollow‐square 3C‐SiC/Si sensor. (A) Response voltage with different airflow velocities, tested at various heat source temperatures. (B) Temperature difference (∆T) between the hot and cold ends at different flow velocities and heat source temperatures. (C) Comparison of the output performance in this work with previous works. (D) Voltage response of the sensor to different airflow frequencies tested at various temperatures. (E) The response time and recovery time of the sensor to the airflow velocity of 2.45 (m/s) at a heat source temperature of 313 K. (F) Voltage variation of the hollow structure to the response of the sensor measured for more than 1000 cycles under an airflow velocity of 2.45 (m/s) and a heat source temperature of 313 K.

The promising improvement of the hollow‐square structure led us to further explore its sensing performance. We first investigated the dependence of voltage variation (Δ*V*)—defined as the thermal voltage difference with and without airflow on varying heat and cooling sources, Figure 4A. The results indicate that the voltage variation increases with either rising airflow velocity or increasing heat source temperature. When the heat source temperature is fixed, increasing the airflow velocity at the cold side enhances the temperature gradient across the device, thereby increasing the output voltage. For instance, at a temperature of 309.5 K, the voltage variation increases from 26.2 µV to approximately 104 µV as the velocity increases from 0.3 to 3 m/s. Similarly, at a fixed airflow velocity, raising the heat source temperature increases the hot‐side temperature, which also enhances the temperature gradient and leads to higher output voltage. For example, at the flow velocity of 2.45 m/s, the voltage variation at a heat source temperature of 333 K is approximately 3.5 times higher than that at 303.5 K.

The dependence of the voltage variation on flow velocity or heat source temperature closely matches the temperature difference between the two SiC electrode areas (Figure 4B), well proving the electrical signal responses to these stimuli. Furthermore, at a temperature gradient of approximately 3 K, the thermalvoltage variation reached approximately 198 µV (Figure 4A). This value is approximately 2.5 times higher than that of 3D‐spacer fabric (PEDOT:PSS/SF) [43], about 1.7 times greater than that of nano fiber yarns (CNT/PEDOT:PSS) [50], and is comparable to that of other thermoelectric materials in previous literature [19, 25, 51, 52]. Figure 4C shows the excellent performance of our sensor compared with previous reports, highlighting its high voltage response under a small temperature gradient. Furthermore, the developed sensor exhibits reliable responsiveness to airflow frequencies ranging from 0.1 to 1 Hz (Figure 4D) under different heat source temperatures, demonstrating its capability to accurately detect different respiratory rate.

Besides, the hollow square 3C‐SiC sensor demonstrates fast response to the airflow. The response time and recovery time of the sensor were evaluated under the same airflow velocity (2.45 m/s), frequency (0.1 Hz), and a temperature of 313 K. Here, the response time and recovery time are defined as the duration that the voltage variation reaches 90% of its maximum value and returns to 10% of its baseline, respectively. As shown in Figure 4E, the sensor exhibits a significant fast response time of approximately 0.9 s, which is comparable to those reported in previous literature (Table S1) [14, 15, 53, 54]. The recovery time is also relatively short (1.4 s). The combined 2.3 s for one cycle suggests a maximum theoretical full‐cycle rate of approximately 26 breaths per minute (bpm) (≈ 0.435 Hz) for the signal to reach maximal values and fully return to baseline. However, our device detects respiratory rate by tracking the peak‐to‐peak periodicity of the output signal (voltage variation ∆*V*). As demonstrated in Figure 4D, the sensor exhibits reliable responsiveness to airflow frequencies ranging from 0.1 to 1 Hz. The frequency of 1 Hz corresponds to a respiratory rate of 60 breathsper minute. Although ∆V decrease slightly at higher airflow frequencies, the sensor successfully produces a distinct, traceable periodic signal at these elevated rates. This confirms that our device is capable of accurately detecting breathing frequencies up to 60 bpm.

Stability is a critical factor in ensuring consistent reliability and quality of sensor performance over time. The stability and robustness of the proposed sensor were further verified through cyclic testing. As depicted in Figure 4F, the stable response behavior was maintained over 1000 cycles when an airflow of 2.45 m/s, frequency of 0.1 Hz, and the heat source of 313 K were applied, demonstrating its excellent long‐term stability. Furthermore, the stability of the devices was evaluated by monitoring the normalized resistance (R/R_0_) at different relative humidity (RH) levels and elevated temperatures. Here R_0_ and R denote the device resistance at the initial state and during measurement. Only negligible changes in R/R_0_ were observed across the RH ranges from 30% to 90%, confirming the device's robustness against moisture (Figure S7A). The device also exhibits high stability in the normalized resistance when exposed to prolonged high‐humidity conditions (RH = 90%; Figure S7B) and various elevated temperature conditions (Figure S7C), demonstrating the excellent long‐term stability of the sensor under high humidity and high‐temperature conditions.

**FIGURE 5:**
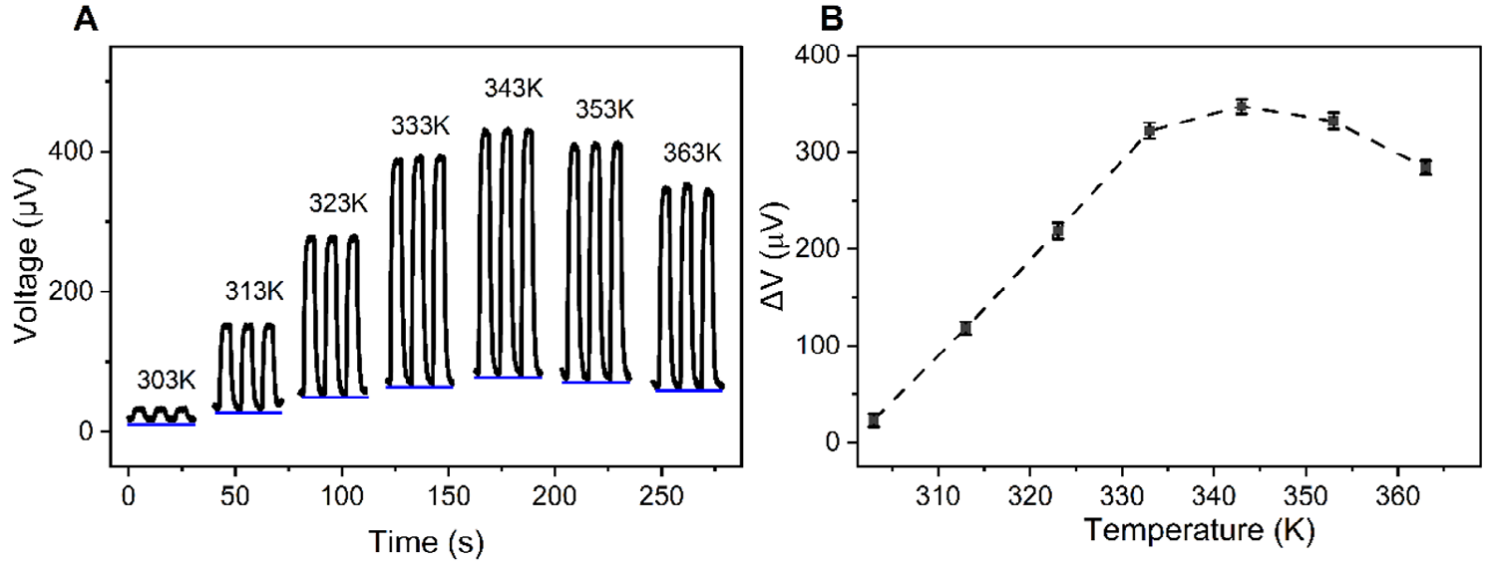
(A) The dynamic response of the sensor with and without airflow velocity of 2.45 (m/s) under different heat source temperatures. (B) Comparison of the voltage variation between low temperature (less than 343 K) and high temperature (greater than 343 K).

Interestingly, we observed that the thermal voltage variation (the voltage difference with and without airflow) exhibits different behavior at temperatures above 343 K, as shown in Figure 5. Figure 5A illustrates the dynamic response of the sensor when  the heater temperature is raised from 303 to 363 K, showing the output voltage with and without airflow (blue lines). Furthermore, the thermal voltage variation tends to decrease at high temperatures (greater than 343 K), Figure 5B. To further investigate this phenomenon, we conducted an experiment to test the sensor's output voltage by steadily increasing the temperature of the heat source under the hot electrode, while the cold electrode was exposed only to the ambient (Figure S8). The thermal voltage initially increases and starts to decrease after the heat source temperature reaches 343 K, even reverses sign at 373 K. We note that the temporary voltage peak (Figure S8 Inset) is caused by the transient thermal effect [55, 56]. When the hot‐side temperature suddenly increases, a temporary temperature gradient peak is established, causing the brief voltage spike, which then decreases to the steady‐state value as heat diffuses through to the cold side. This observed voltage behavior at elevated temperature is particularly important for further developing sensor applications in high‐temperature environments, such as deep‐mine or firefighting conditions. We propose a mechanism for this observation, which can serve as a foundation for future research aimed at optimizing and improving sensor performance in these conditions. The reversal sign of thermal voltage can be explained as follows.

From Equation (1), the sign of the thermal voltage depends on the sign of the Seebeck coefficient of the material. For the 3C‐SiC/Si heterojunction, the Seebeck coefficient (*S*_h_) can be approximated using a simple two‐parallel conduction model [57, 58].

(3) $$S_{h}=\frac{S_{3C-SiC}\sigma_{3C-SiC}+S_{Si}\sigma_{Si}}{\sigma_{3C-SiC}+\sigma_{Si}}=S_{3C-SiC}\;\left(\frac{1}{1+\alpha }\right)+S_{Si}\left(\frac{1}{1/\alpha +1}\right)$$

where *S*_3*C* − *SiC*_, *S_Si_*,σ_3*C* − *SiC*_, σ_*Si*_ are the Seebeck coefficients and the conductivity of the 3C‐SiC and Si layers, respectively. α is σ_*Si*_/σ_3*C* − *SiC*_.

At low temperatures, the conductivity of the Si layer is much lower than that of the 3C‐SiC layer in the 3C‐SiC/Si heterostructure (Figure S9) [34]. Therefore, σ_3*C* − *SiC*_ ≫ σ_*Si*_ and α→0; the Seebeck coefficient of the heterojunction (*S*_h_) is dominated by the n‐type 3C‐SiC layer [58]. As a result, the sign of thermal voltage is governed by the sign of *S*_3*C* − *SiC*._ However, at elevated temperatures, the leakage current into Si increases, while the current through 3C‐SiC decreases (Figure S9). Therefore, we can consider that the conductivity of the Si component increases more strongly than the SiC layer (α increases). As the α increases, the SiC component of *S*_h_ decreases while the Si component of *S*_h_ goes up. In other words, based on the parallel model, the *S*_h_ of the heterojunction is considered to have a transition from the dominance of 3C‐SiC to the dominance of Si.

It is commonly known that under a temperature difference, electrons will move from the hot side to the cold side for n‐type semiconductors like n‐type 3C‐SiC, while holes will move from the hot side to the cold side for p‐type semiconductors like p‐type Si [42, 59, 60, 61]. Therefore, the sign of the Seebeck coefficient of n‐type 3C‐SiC is opposite to that of p‐type Si. As a result, when the temperature increases, *S*_h_ shows the reversed sign due to the transition from the dominance of SiC to the dominance of Si.

**FIGURE 6:**
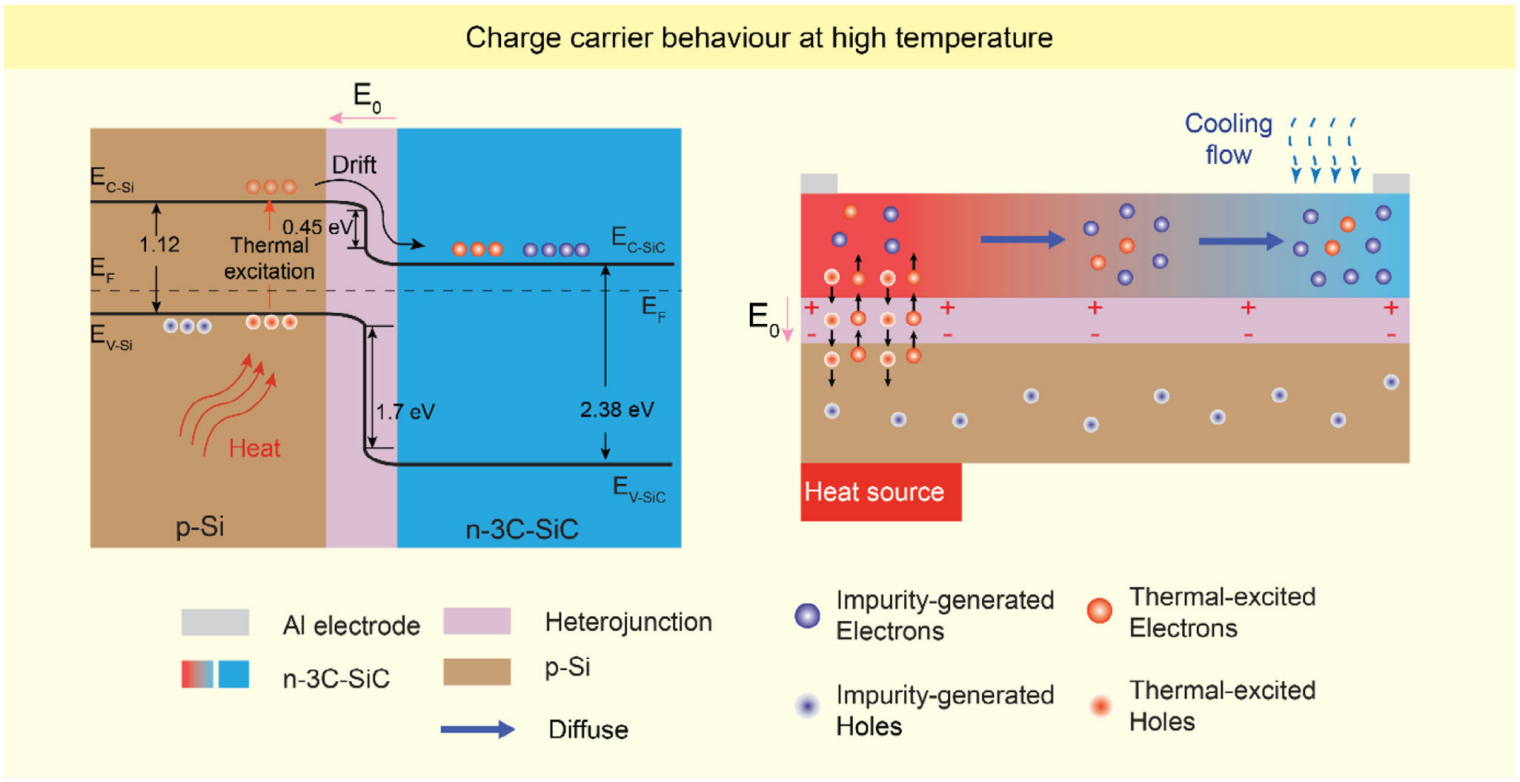
Charge carrier behavior under high temperature. (Left) Thermal‐excited electrons in Si move to 3C‐SiC due to the built‐in electric field. (Right) Illustration of the electron distribution between the hot and cold sides under high temperatures.

In terms of carrier transport mechanism, at high temperatures, the heterojunction plays a role in altering the thermoelectric behavior of 3C‐SiC/Si. Thermal‐generated electrons in Si substrates can move toward the Si/3C‐SiC junction, in which they are swept to 3C‐SiC by the built‐in electric field pointing from 3C‐SiC to Si (Figure 6, left; Figure S10) [62]. The current leakage to the Si substrate at high temperature in Figure S9 is evidence for such electron movement [34]. Therefore, the hot side should have more thermal‐generated electrons than the cold side. The representative in Figure S10 only shows the phenomenon on the hot side for simplification. Furthermore, electron mobility impacts the diffusion of electrons from the hot to the cold side via the diffusion coefficient *D*_e_ = µ_e_(*kT*/e) [44], in which µ_e_ is the electron mobility, *k* is the Boltzmann constant, *T* is the absolute temperature, and *e* is the elementary charge. In n‐type 3C‐SiC, electron mobility was reported to decrease with temperature [63]. The *r* factor (in $\mu_{e}∼T^{r}$) is considered to be from −3/2 to 0, indicating the presence of phonon scattering [44, 63]. Therefore, it becomes difficult for electrons to move from the hot side to the cold side at high temperatures. The combination of these two factors contributes to a larger number of electrons on the hot side compared to the cold side (Figure S10), resulting in a large thermal voltage under higher temperatures (above 373 K) with the inverse sign.

Moreover, the turning point occurs at around 343 K (Figure 5; Figure S8). This indicates a transition from the conventional Seebeck effect of n‐type semiconductor to the combined effect of Si substrate, 3C‐SiC/Si heterojunction, and temperature‐dependent electron mobility of 3C‐SiC. Since the sign of the thermal voltage and the voltage variation is not changed around that point, the cold side still presents more electrons than the hot side; however, the difference in electron concentration between the two is illustrated to be relatively modest (Figure 6, right). These results are expected to facilitate the development of thermoelectric devices based on the heterojunction of semiconductor applied in elevated temperatures (such as deep mines or firefighting).

For healthcare applications, the biocompatibility of the functional materials plays a crucial role in mitigating the risk and ensuring long‐term user safety. The biocompatibility of 3C‐SiC membranes has been tested in our previous study through in vitro cell cultures [35], which confirmed that the viability of Human Dermal Fibroblast (HDF) and Mouse Fibroblast (MF) cells on the nanomembrane remained comparable to control samples after 72 h (Figure S11A). Furthermore, we conducted an irritation test on a healthy volunteer by attaching a 3C‐SiC/Si chip to his forearm under a medical bandage for 24 h. The application site was subsequently examined at 24 and 24 h after the chip was removed. As shown in Figure S11B,C, no adverse skin responses, such as a rash or other skin change, were observed, demonstrating the safety to human skin. These results indicate that the 3C‐SiC/Si material exhibits good biocompatibility and is safe for human use.

### 2.3 Demonstration of the Self‐Powered Thermoelectric 3C‐SiC/Si Sensor for Respiration Monitoring

**FIGURE 7:**
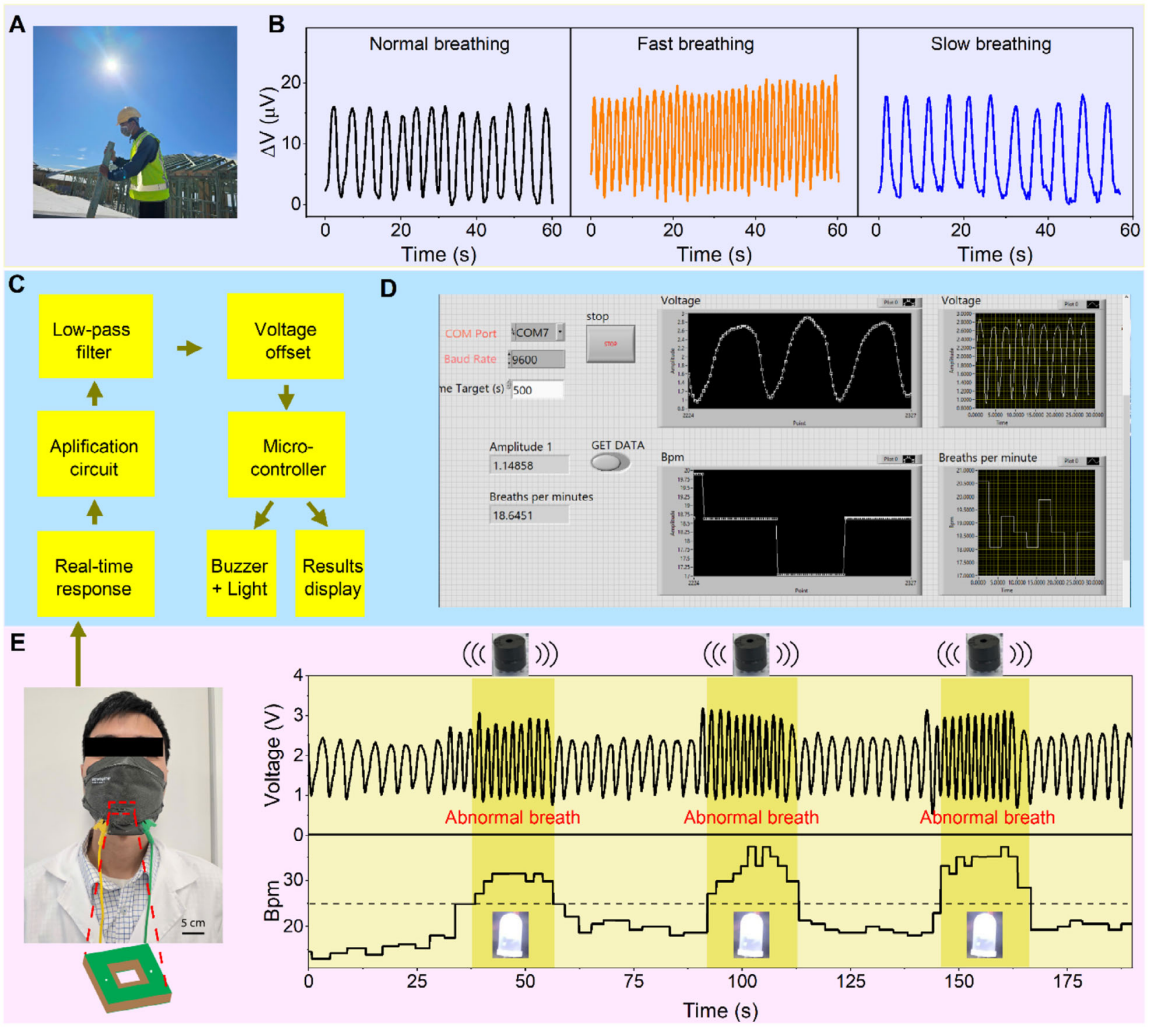
Demonstration of the self‐powered 3C‐SiC/Si sensor for real‐time respiratory monitoring. (A) Image of a construction worker wearing a mask during working under high‐temperature environment, demonstrating that the fabricated 3C‐SiC sensor can be integrated into a facial mask. One end of the sensor was exposed to the hot ambient air, and the other end was positioned near the breathing input to monitor respiratory rate. (B) Recorded respiration signals, including normal, fast, and slow breathing in the hot environment. (C) Schematic illustration of the 3C‐SiC/Si self‐powered respiratory sensor and its warning system. (D) Demonstration of real‐time monitoring of respiratory rate recorded from the system. (E) Illustration of a volunteer wearing the functional mask during breathing. (F) The breathing signal after amplification. The buzzer and Light‐ Emitting Diode (LED) will be activated to alarm the wearer when his breathing rate suddenly increases over 25 breaths per minute.

Monitoring the breathing rate of workers in hot environmental conditions can potentially enhance workforce productivity, safety, and overall well‐being [6, 64]. For example, an abnormally rapid respiratory rate may serve as an early indicator of heat‐related stress or overexertion, prompting timely intervention to protect workers' health [65]. In outdoor high‐temperature conditions, the ambient temperature often exceeds the average human body temperature (>309.5 K), which can heat the sensor on one side. On the other hand, exhaled airflow, which is close to body temperature, can locally cool the temperature on the other side of the sensor. This creates a temperature gradient across the sensor, resulting in a thermally generated voltage that can be used to detect the breathing rate. Therefore, the proposed sensor can be integrated into a face mask to continuously monitor of respiratory rate of outdoor workers in real time, without requiring an external power source (Figure 7A). To achieve this, one end of the sensor was placed outside of the mask, directly exposed to the hot environment, serving as the hot end. The other end was positioned inside the face mask, near the nostrils, to serve as the cold end, utilizing the cooling effect of breathing flow. The hollow square structure does not obstruct the main filter area or interfere the airflow pathway. Therefore, this integration system maintains the expected level of breathability and comfort worker in outdoor settings.

To simulate a high‐temperature environment, a heater was activated to raise the surrounding temperature to approximately 313 K. A participant wearing the mask integrated with the hollow‐window 3C‐SiC sensor performed different breathing modes in these conditions. The sensor effectively captured the changes in the respiratory rate of the participant under various conditions, including normal, fast, and slow breathing. Since different health conditions result in distinct respiratory rates [66], the frequency of voltage variation follows correspondingly. As shown in Figure 7B, the sensor can distinguish between normal breathing (frequency of 0.23 Hz), fast breathing (frequency of 0.58 Hz), and slow breathing (frequency of 0.18 Hz).

In addition to hot environments, the sensor also functions effectively at room temperature, which is typically lower than body temperature. In this scenario, the sensor can be integrated into the mask in the same manner. However, the ambient‐exposed end now becomes the cold end, while the end near the nostrils, warmed by exhaled air, serves as the hot end (Figure S12A). The results presented in Figure S12B–D clearly indicate the capability of the sensor for monitoring different breathing conditions, including resting state, post exercise, and holding breath. Furthermore, the successful 1 h continuous monitoring while wearing the mask (Figure S13) further confirms the comfortable wearing during prolonged use and the capability of continuous respiratory monitoring of our device.

Furthermore, abnormal breathing rate is considered a predictive sign for individual health concerns [7, 10]. Therefore, we demonstrated an alarm system designed to alert users to abnormal breathing. The system consists of a self‐powered 3C‐SiC sensor, a signal acquisition, a processing unit (Arduino microcontroller), a display using LabVIEW software, and an alert module consisting of a buzzer and a Light‐Emitting Diode (LED) as indicators (Figure 7C). As displayed in Figure 7D, the shape of the breathing pattern was recorded by the software, from which the respiratory rate was extracted.

The user wearing this alarm system (Figure 7E) has the benefits of visualizing his breathing patterns, capturing the changes in the pattern, as well as being alerted to abnormal breathing conditions, such as a sudden rise in breathing rate. Sudden increase in breathing rate may be considered as an alarm for acute asthma, COPD [67] or an early sign for heat‐related stress [65]. We conducted a test by asking the volunteer to simulate an abnormal breathing by increasing his respiratory rate to over 25 breaths per minute on three consecutive occasions. In response, the buzzer will sound an alarm, and the light will turn on (Figure 7F; Video S1). Moreover, respiratory arrest poses a significant life‐threatening risk if not identified promptly. During normal breathing, the 3C‐SiC sensor produces continuous electrical signals. However, when the user suddenly holds his breath, the signal is interrupted. If the duration of holding breath exceeds 10 s, the system triggers an alarm with both visual and audible alerts. Additionally, as the subject begins to breathe rapidly after holding his breath, typically to compensate for oxygen deprivation, the system is also activated (Figure S14 and Video S2). These results demonstrate the feasibility of the proposed sensor for continuous respiratory monitoring and the early detection of symptoms associated with asthma or sleep apnea. It also serves as a preventive safety measure to alert construction workers or individuals in high‐risk environments, allowing them to take timely breaks and reduce workloads in response to heat stress. Future work could explore on miniaturization and wireless integration. The miniaturized wireless sensing system would be a thin, flexible thermoelectric heterojunction sensor and a flexible wearable circuitry system built on flexible and stretchable substrates such as PDMS. This circuit, typically comprising mini microcontrollers with supported wireless communication and analog‐to‐digital converter, signal amplifications and filters, and other common electronics such as resistors and capacitors, would be designed to process the low‐voltage Seebeck output. The PDMS substrates, microcontrollers, and electronics can resist high temperature (up to or even above 100°C), Therefore, it is feasible to explore miniaturization and wireless integration to improve applicability across diverse scenarios.

Furthermore, the unique behavior, reversed sign of the output voltage, of the 3C‐SiC/Si heterojunction at elevated temperature, is primarily important for sensor application in detecting hazardous safety zones in firefighting conditions. For demonstration, we mounted the 3C‐SiC/Si sensor on a protective enclosure designed, which can be embedded on the firefighting gear. One side was exposed to a simulated hot environment, while the other side remained on the protected interior side. Controlled hot airflow (Micro hot air gun 8858IV, YIHUA) was used to simulate environmental heat flow at different temperatures. As shown in Figure S15, when one end of the sensor is exposed to heat flow with a temperature of approximately 339 K, it generates a positive voltage. However, the voltage reverses sign once the temperature reaches 378 K or higher. In firefighting scenarios, this shift from a positive to a negative sign can be exploited as a critical threshold to warn firefighters to move away from the hot spots timely, thereby preventing life‐threatening injuries.

## 3 Conclusion

In conclusion, a hollow‐square self‐powered 3C‐SiC/Si is proposed for real‐time monitoring of human respiration rate, aiming to provide early warning of abnormal health conditions for outdoor workers under hot environments. This hollow structure enables manipulation of thermal transport, while the heterojunction regulates the transport of thermally generated charge carriers. These innovations yield a sensitivity 3.5 times higher than that of conventional designs and provide high stability under complex temperature–humidity–flow conditions. Furthermore, as the temperature rises, the dominant mechanism shifts from the conventional Seebeck effect in n‐type semiconductors to a heterojunction‐driven effect, providing a crucial foundation for the development of self‐powered 3C‐SiC/Si‐based sensor for application at elevated temperatures, such as firefighting conditions. A practical application scenario is demonstrated by integrating the sensor into a functional facemask, with one end exposed to the hot ambience and the other positioned near the nostrils inside the mask. The results indicate the sensor can effectively detect different respiratory rates, including abnormal patterns. Furthermore, by integrating the sensor into an alarm system, users can be alerted to abnormal respiration conditions. Therefore, this study presents a promising step toward developing self‐powered devices for monitoring physical health at high temperature environments and providing timely alerts.

## 4 Experimental Section

### 4.1 Fabrication Process

The 3C‐SiC/Si wafer was fabricated using LPCVD at a temperature of 1000°C. Propene and silane precursors were used via low pressure to provide Si and C sources in the 3C‐SiC growth process. After the growth process, a photoresist layer was coated on 3C‐SiC by spin‐coating technique at a speed of 4000 rpm, followed by soft‐bake at 100°C for 60 s. Next, the photoresist was exposed to ultraviolet light and subsequently developed for 30 s in AZ726 MIF, followed by rinse with the deionized (DI) water. The sample was then hard baked at 120°C for 2 min prior to dry etching. Dry etching SiC surface using inductive coupled plasma (ICP) to open square windows, with the etching rate of SiC is approximately 100 nm/min. Strip photoresist using O_2_ plasma for 25 min. After that, wet etching the Si layer until the hollow is formed using KOH (30% wt) then decontaminated KOH‐etched sample using RCA cleaning. Finally, the SiC membrane was removed to create a hollow‐square 3C‐SiC/Si structure. This sample was then cleaned to avoid contamination before electrical interconnection. Direct Al wire bonding method was employed to make electrical connections on the 3C‐SiC, as presented in our previous work [68]. The carrier concentration of the n‐SiC layer and the p‐Si substrate were around 10^19^ and 5 × 10^14^ cm^−3^.

### 4.2 Material Characterization

The surface and structural morphology of the 3C‐SiC grown on the Si wafer was investigated using atomic force microscopy (AFM, Asylum Research Cypher), and JEOL NeoScope JCM ‐7000 Benchtop SEM. Moreover, Raman spectra were measured using Renishaw Virsa Raman Analyzer (SC 100) with a laser excitation wavelength of 532 nm. The long‐term humidity stability test was controlled by using a humidity chamber (Linkam MFS350). The hot plate (LINKAM HFS600‐PB4) was employed to examine the stability of the device under elevated temperatures.

### 4.3 Sensing Characterization

To investigate the performance of the device, we constructed a custom‐built testing platform. One end of the devices was deposited on a Linkam MFS350 heating stage. The temperature of the hot plate was precisely controlled using the LINK software connected to the MFS350. The distance from the hot plate to the cold electrode was controlled at a constant distance of 2 mm. The temperature of the hot electrode was controlled by setting the temperature of the heating stage increase from 303 to 343 K. The temperature of the hot and the cold electrode the hot and cold electrodes was measured by RS PRO Digital Thermometer. The airflow was generated using a Jun‐Air compressor connected to a pressure controller (ElveFlow MK3+), with an inner tube diameter of 2.0 mm. The distance between the airflow tube and the cold electrode was maintained at 1 cm, with the tube directed toward the cold electrode area. Air at room temperature (25°C) was supplied, with flow rates controlled from 0.2 to 3 m/s and flow frequencies ranging from 0.1 to 1 Hz. All measurements were performed in dark conditions at a room temperature of 25°C.

### 4.4 Sensing Performance Test of the Sensor

The real‐time electrical signals were collected by a Source Meter (Keithley 2450).

### 4.5 Informed Consent Statement

All experiments related to human breath monitoring were conducted following applicable laws and institutional guidelines and were approved by the Human Ethics Committee (HREC) of the University of Southern Queensland (Approval Number: ETH2025‐0427). In accordance with ethical requirements, informed written consent was obtained from the participant prior to participation. The participant also provided written consent permitting the use and publication of his photograph in this manuscript.

## Funding

The Queensland Department of Environment, Science and Innovation under the Quantum 2032 Challenge Program (Grant No. Q2032010) and the Australian Research Council through grants DP220101252 and DP240102230.

## Conflicts of Interest

The authors declare no conflicts of interest.

## Supporting information




**Supporting File**: smll72469‐sup‐0001‐SuppMat.docx.

**Supporting File 1**: smll72469‐sup‐0002‐VideoS1.mov.

**Supporting File 2**: smll72469‐sup‐0003‐VideoS2.mov.

## Data Availability

Data will be available on request.
