# Supplementary material for: A Sensitive Thermoelectric Respiratory Sensor Using a Hollow‐Square Structure of Cubic Silicon Carbide‐Based Heterojunction
Source: Small. 2026 Jan 20;22(16):e10634. doi: 10.1002/smll.202510634 (PMC12994552; doi:10.1002/smll.202510634)
Supplement: Supplementary file 1 — Supporting File: smll72469‐sup‐0001‐SuppMat.docx. [file SMLL-22-e10634-s002.docx]

Supporting Information

# **A Sensitive Thermoelectric Respiratory Sensor Using a Hollow-Square Structure of Cubic Silicon Carbide-Based Heterojunction**

*Thi Lap Tran^*^, Duy Van Nguyen, The Lai Khanh, Thien Hoang, Toan Trong Tran , Pingan Song, Nam-Trung Nguyen, Dzung Viet Dao, John Bell, Ravinesh C Deo, and Toan Dinh^*^*


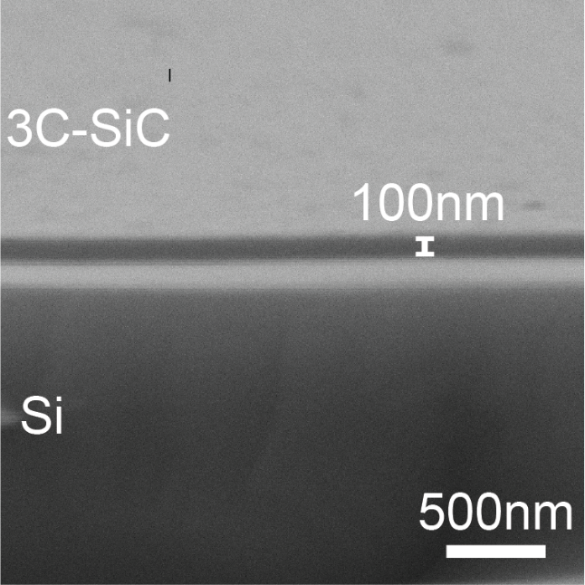


**Figure S1.** SEM image of 3C-SiC/Si Heterojunction, showing an the 100-nm thick 3C-SiC layer deposited on the Si substrate


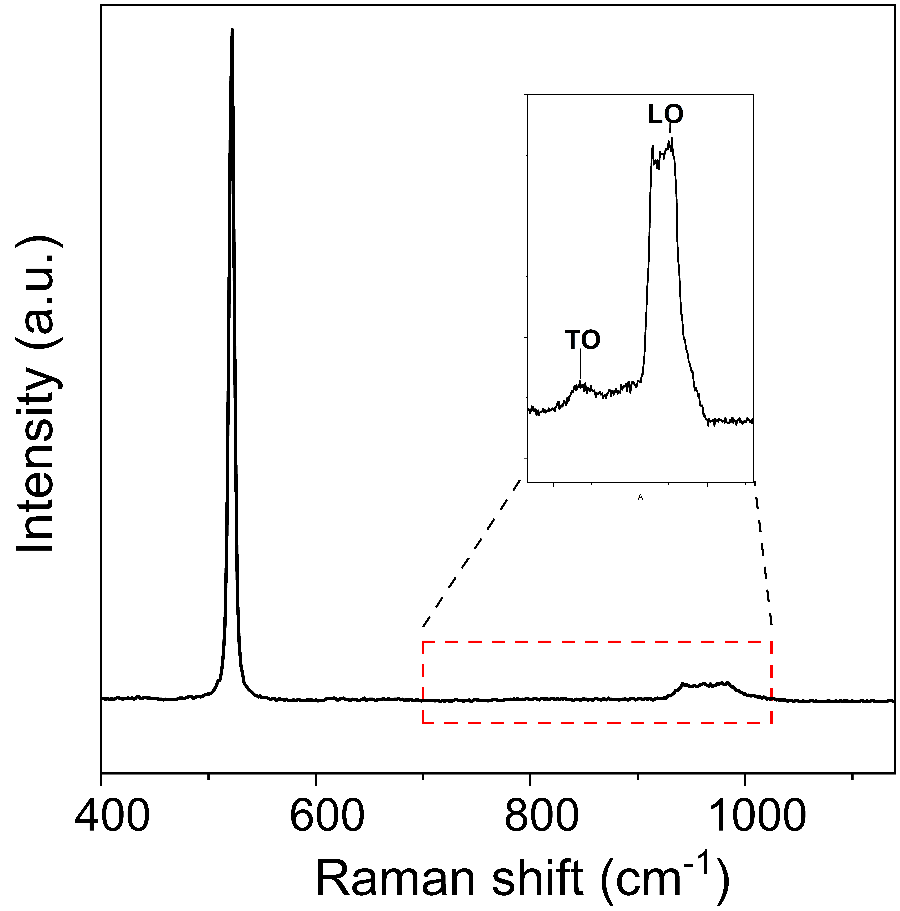


**Figure S2**: Raman spectrum of 3C-SiC/Si in range of 400 –1150cm^-1^


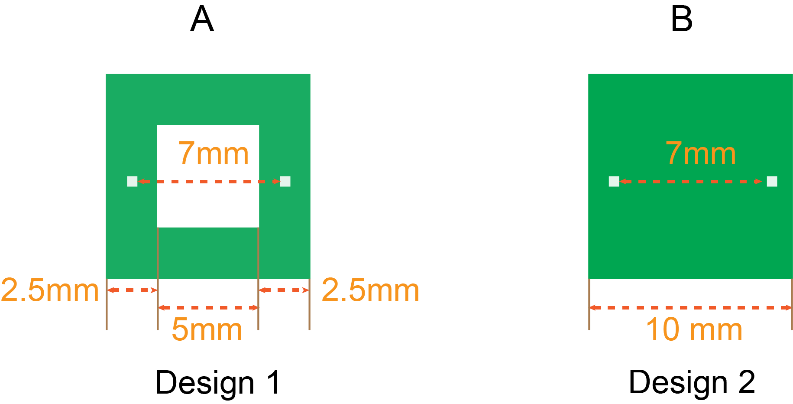


**Figure S3.**  Dimensions of the devices in top view (not for scale). (A) Design 1 and (B) Design 2. The white dot indicates the position of the electrode.


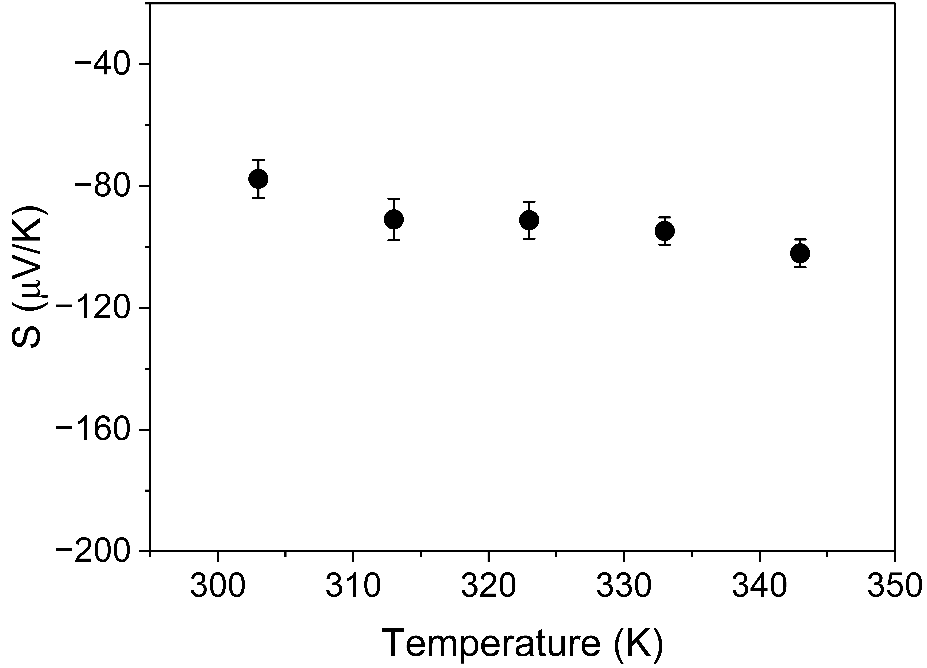


**Figure S4**. The Seebeck coefficient of 3C-SiC/Si corresponds to the temperature of the hot plate under hot electrode.The measurement was carried out three time for each condition.


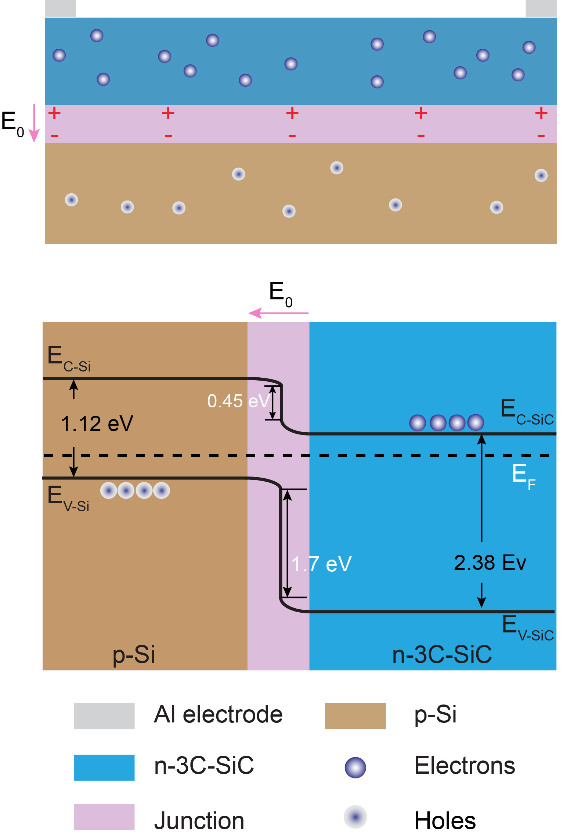


**Figure S5**. Band diagram of 3C-SiC/Si heterojunction when n-type 3C-SiC and p-type Si are in contact.


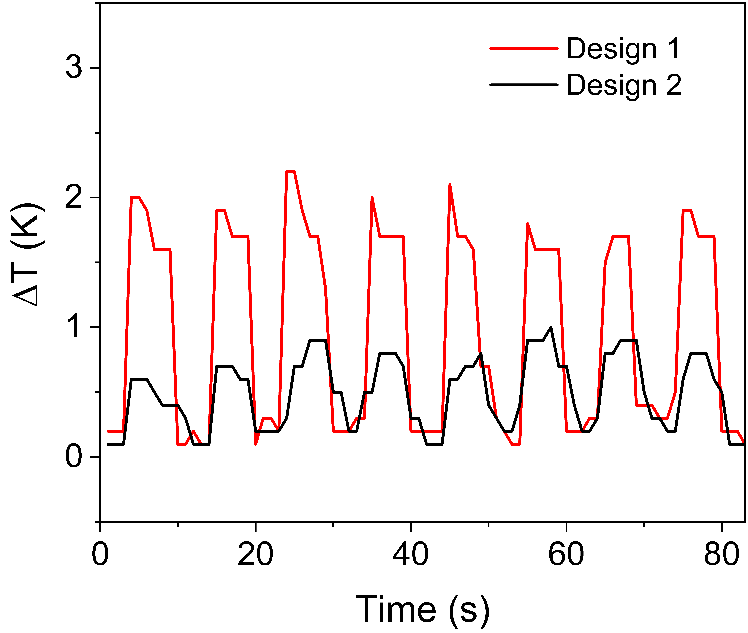


**Figure S6.** Temperature differences between the hot and cold ends of Design 1 and Design 2 under a heate source of 313 K , and airflow velocity of 2.45 (m/s), indicating that Design 1 exhibits large temperature gradient between the two electrodes compared to Design 2.


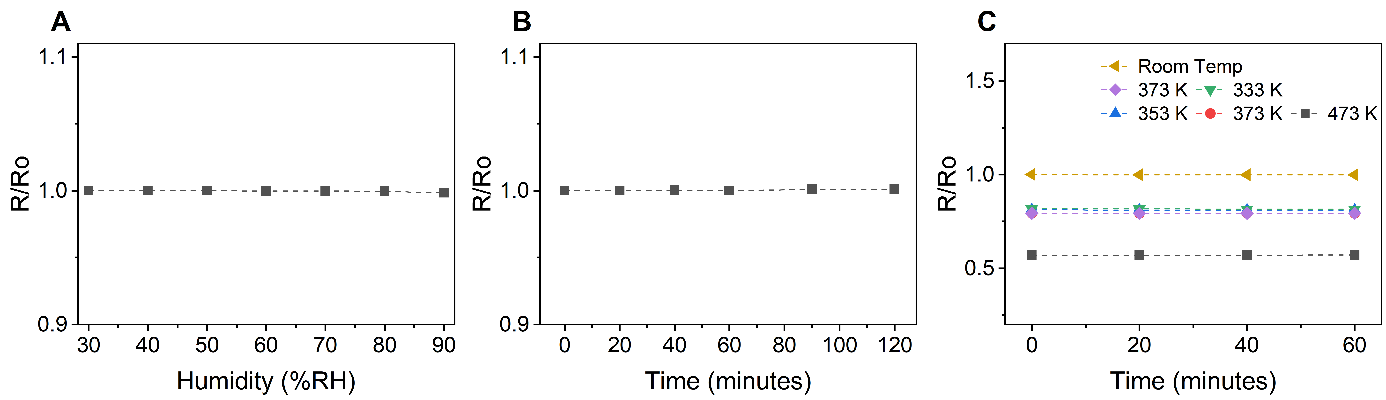


**Figure S7**. Stability of the device under high humidity and temperature condition. (A) Normalized resistance of the sensor under related humidity (% RH) ranges from 30 to 90% at a constant temperature of 303 K. (B) Normalized resistance of the sensor monitored continuously over 2 hours at 90% RH. (C) Normalized resistance recorded for an hour at various temperatures.

For humidity stability, the device was placed in a humidity chamber (Linkam MFS350) maintained at a temperature of 30 ± 0.4 °C, with the relative humidity (RH) ranges from 30% to 90%. The normalized resistance (R/R_0_) was recorded at each stage, in which R_0_ is the resistance at baseline condition (30% RH). Additionally, when the device was held continuously at 90% RH for 2 hours, minimal change was observed in the normalized resistance (**Figure S7A-B**), confirming the excellent long-term stability of our device in high-humidity conditions.

For thermal stability test, the device was placed on a hot plate (LINKAM HFS600-PB4), and its temperature was set to increase from room temperature to 333 K, 353 K, 373 K, and 473 K respectively and each temeprature was maintained for an hour. No noticeable change in the normalized resistance was observed at any of the tested temperatures (Figure S7C). Here R_0_ and R denote the device resistance at room temperature and during measurement. These findings demonstrate the excellent long-term stability of the device at elevated temperatures.


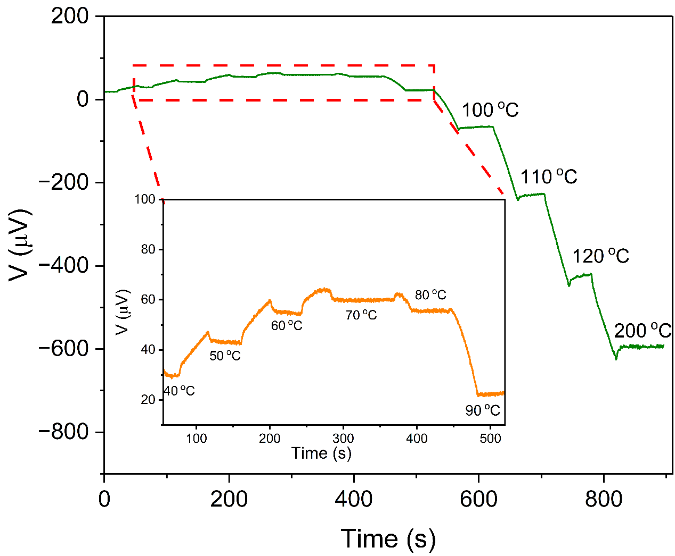


**Figure S8.** Voltage response of the of the 3C-SiC/Si with the increase of the temperature in the hot electrode, the cold electrode is exposed to the ambient temperature without air flow.


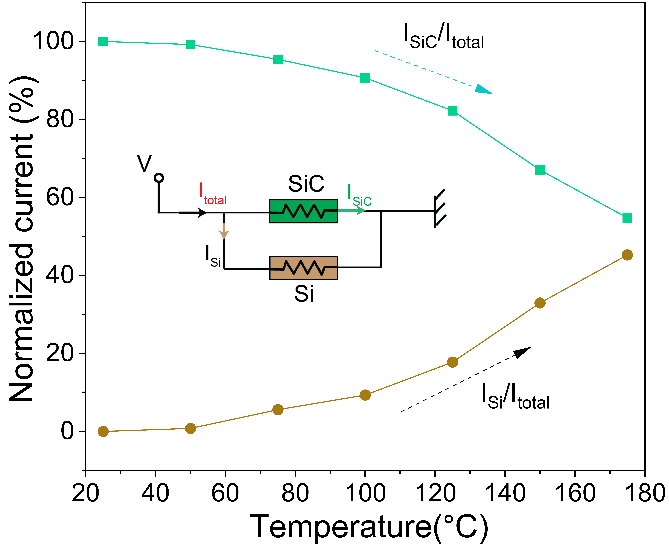


**Figure S9**. Electrical current flow in the 3C-SiC/Si heterojunction Reproduced with permission. Ref. [1] Copyright 2018, WILEY.


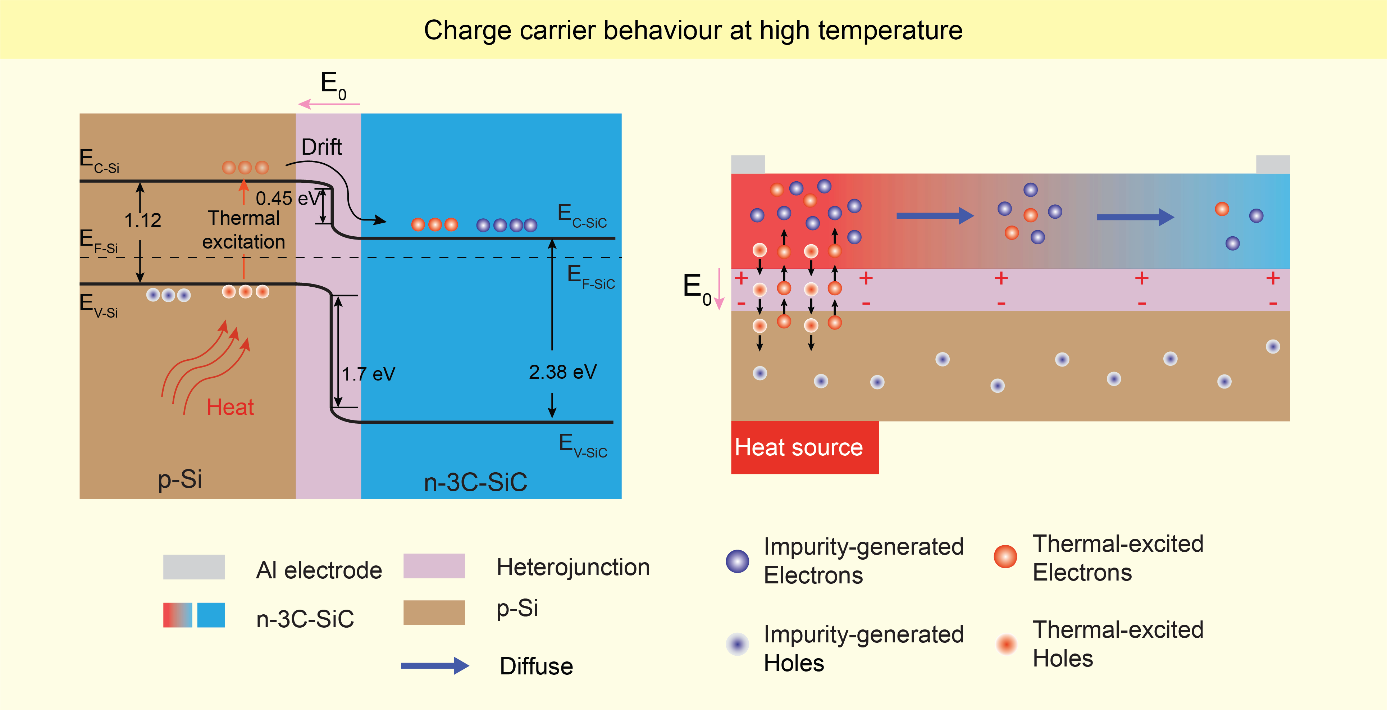


**Figure S10.** Charge carrier behaviour under high temperature (greater than 373 K). (Left) Thermal-excited electrons in Si move to 3C-SiC due to the built-in electric field. (Right) Illustration of the electron distribution between hot and cold side under high temperatures.


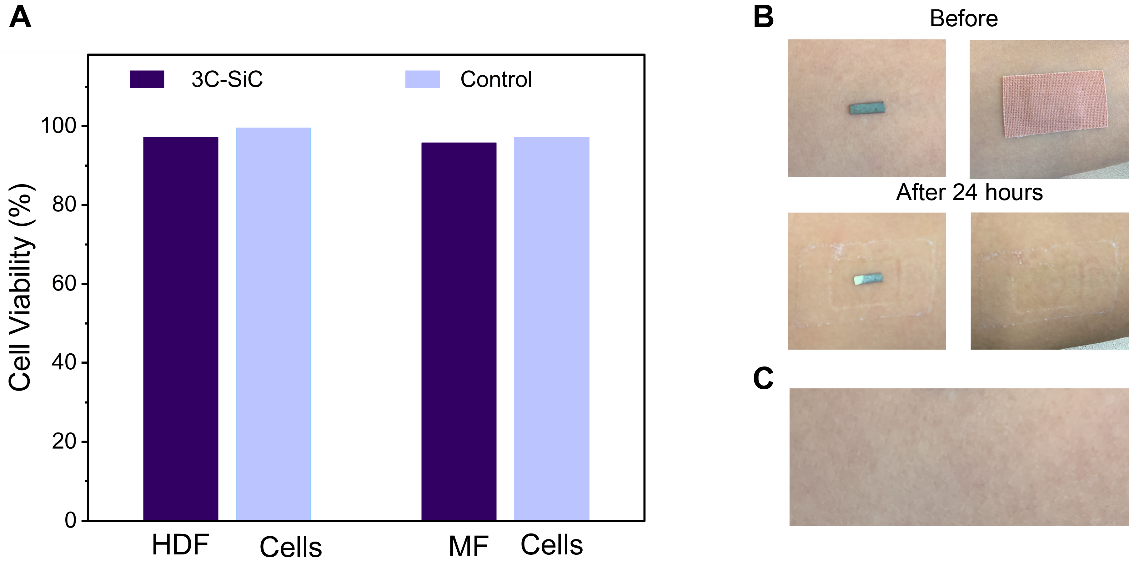


**Figure S11.** (A) Trypan blue (TB) exclusion test shows no significant difference in percentage of viable cells work Reproduce with permission. Ref [2] Copyright 2019, ACS Nano. (B) Image of the volunteer’s skin showing the contact area (top, 3C-SiC/Si attached) and immediately after removing the chip (bottom) following 24 hours of wear. (C) Image of the skin contact area 24 hours after the 3C-SiC/Si chip was removed, confirming the absence delayed irritation.


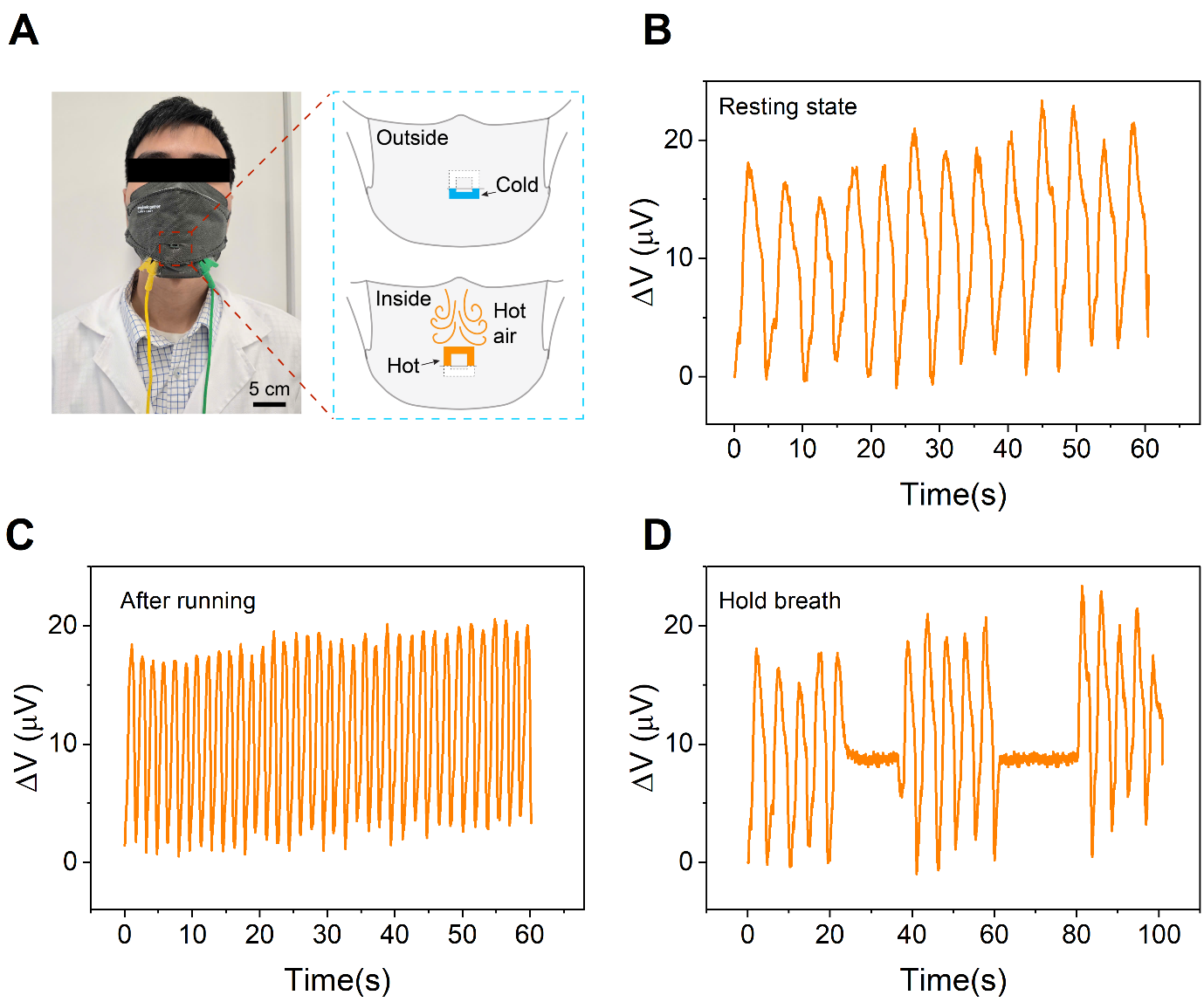


**Figure S12.** (A) Physical image of the 3C-SiC sensor integrated into a mask for respiratory monitoring. One side of the sensor is positioned close to the nasal area, serving as the hot end, while the other is exposed to ambient environment, acting as the cold end. (B-D) Respiratory signals recorded from 3C-SiC self-powered thermoelectric sensor in resting, after running and holding breath.

In room temperature, because of the temperature of exhaled air is higher than the ambient temperature, the 3C-SiC sensor can be used to track respiratory rate without the need for an external power source. **Figure S12A** illustrates the self-powered 3C-SiC sensor integrated into a mask. One end was placed inside the mask, which was exposed to warm exhaled air and served as the hot end. The other end was exposed to ambient environment, serving as the cold end. During breathing, the warm exhaled air acts as a heat source, creating a temperature gradient between the two ends, subsequently generating a voltage signal. As shown in **Figure** S12B-D, the sensor was utilized to monitor respiratory rate of a volunteer under various conditions, including resting, post-exercise, and holding breath. Since different body postures and health conditions result in distinct respiratory rate [3, 4], the respiratory rate changes correspondingly, leading to variations in the generated voltage.


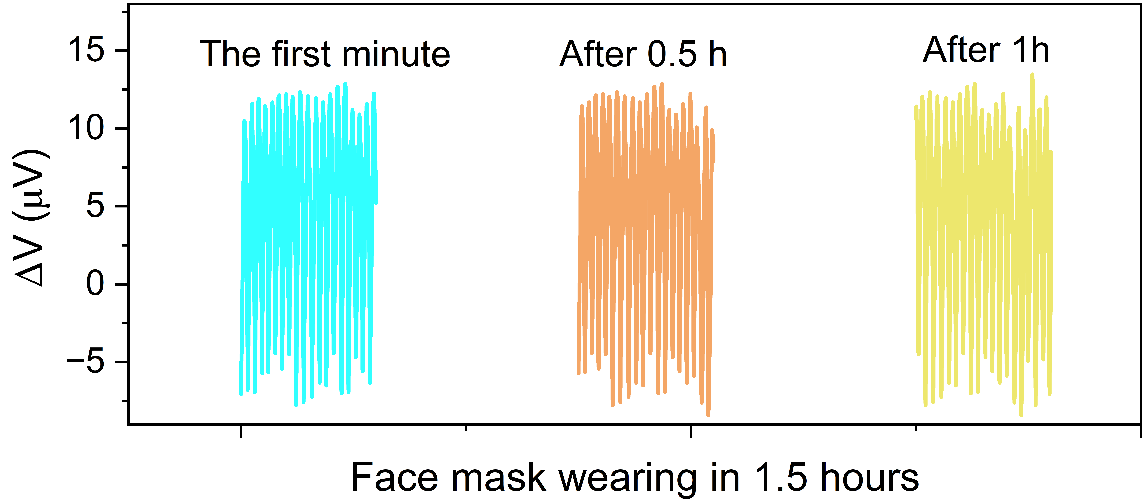


**Figure S13.** Sensor response to normal breathing, measured at 1-minute intervals over 1 hour while wearing a facemask, demonstrating the comfortable wearing and stability of the proposed sensor.


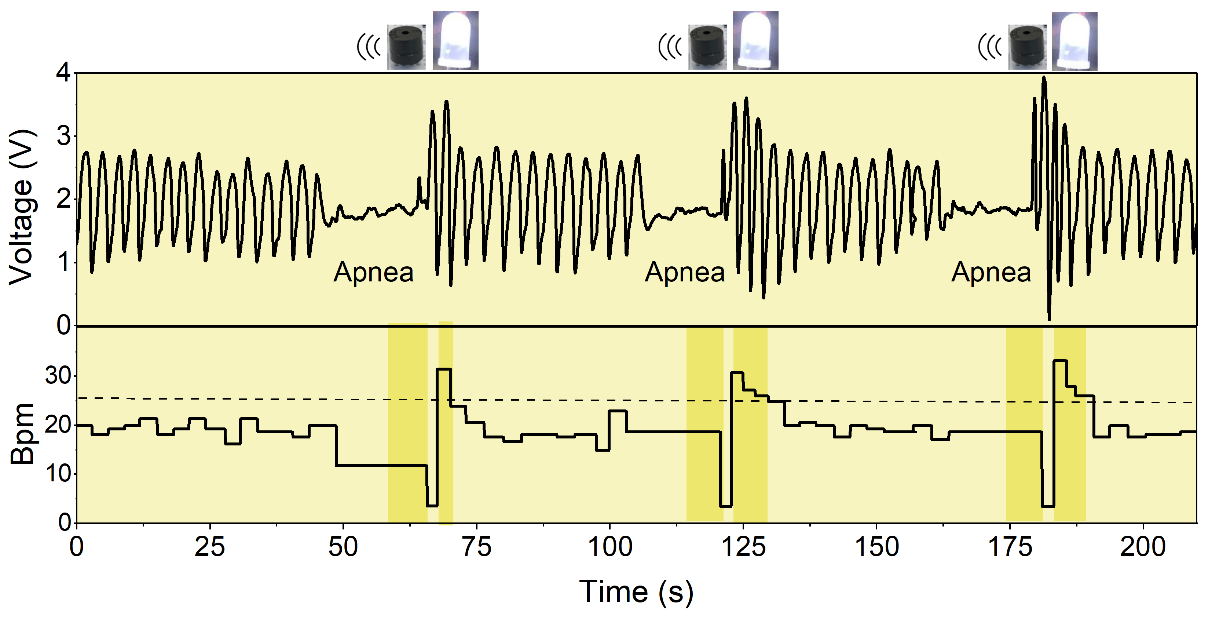


**Figure S14.** Real-time breathing signals when a volunteer simulated apnear symtom after amplified, with a trigger alarm and active light.


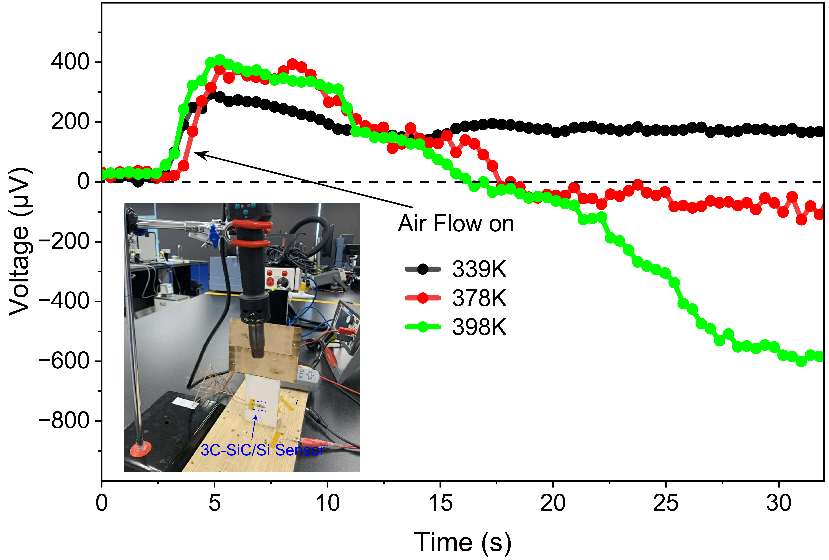


**Figure S15.** Demonstration of the use of 3C-SiC/Si with the ability to detect a threshold temperature to potentially warn firefighters of hot sports. The generated voltage shows a reversed sign at simulated temperatures higher than 378K.

**Table S1**: Comparison Seebeck coefficient and performance of our work with previous literatures.

| Materials | S (µVK^-1^) at room Temp | Temperature(K) | Response time (s) | Application | Ref. |
| --- | --- | --- | --- | --- | --- |
| CNTelectrospray on poly(latctic acid) (PLA) | 62.9 | NA | NA | Breath monitoring | [5] |
| Cotton Fiber | 20 | NA | NA | Temperature sensor | [6] |
| PEDOT: PSS/SF  (3D-Spacer fabrics) | 23 | 0-100 | 1s | Temperature sensor | [7] |
| (PEDOT:PSS)/CNT/WPU | 31 | NA | 0.7s | Temperature sensor | [8] |
| Sb_2_Te_3_+ Bi_2_Te_3_ | (-154.3)/  (-121.5) | 330-420 | 8ms | Breath sensor | [9] |
| MXene/CNT/PEDOT: PSS | -9.8 | NA | NA | Breath sensor | [10] |
| CNT/PEDOT:PSS(nano fiber yarns) | 43 | 297 | 0.55s | Breath sensor | [11] |
| (PEDOT:PSS)/CNT | 35.9 | NA | NA | Temperature sensor | [12] |
| Graphite on paper substrate | NA | NA | 1.4 | Breath monitoring | [13] |
| Silk/CNTs | NA | NA | 1.3 | Airflow monitoring | [14] |
| Graphene/single-wall nanotubes-Ecoflex membrane | NA | NA | 1.04 | Airflow monitoring | [15] |
| β-SiC (n-type Poly) | -10 to -20 | 298-573 | NA | Temperature sensing | [16] |
| β-SiC (n-type) | -50 to -150 | 673-973 | NA | Temperature sensing | [17] |
| β-SiC nanowires | -40 to -65 | 180-370 | NA | Temperature sensing | [18] |
| β-SiC (Sintered SiC) | -111 | 300-400 | NA | Self-cooling device | [19] |
| **n-3C-SiC/p-Si** | **-77 to -102** | **303- 343** | **0.9** | **Breath montoring** | **This work** |

**Reference**s

[1] T. Dinh, H. P. Phan, N. Kashaninejad, T. K. Nguyen, D. V. Dao, and N. T. Nguyen, "An on‐chip SiC MEMS device with integrated heating, sensing, and microfluidic cooling systems," *Advanced materials interfaces,* vol. 5, no. 20, p. 1800764, 2018, doi: https://doi.org/10.1002/admi.201800764.

[2] H.-P. Phan *et al.*, "Long-lived, transferred crystalline silicon carbide nanomembranes for implantable flexible electronics," *ACS nano,* vol. 13, no. 10, pp. 11572-11581, 2019, doi: https://doi.org/10.1021/acsnano.9b05168.

[3] A. Silvani *et al.*, "Physiological mechanisms mediating the coupling between heart period and arterial pressure in response to postural changes in humans," *Frontiers in physiology,* vol. 8, p. 163, 2017, doi: https://doi.org/10.3389/fphys.2017.00163.

[4] S. Mirjalali, S. Peng, Z. Fang, C. H. Wang, and S. Wu, "Wearable Sensors for Remote Health Monitoring: Potential Applications for Early Diagnosis of Covid‐19," *Advanced materials technologies,* vol. 7, no. 1, p. 2100545, 2022, doi: https://doi.org/10.1002/admt.202100545.

[5] S. Liu, M. Zhang, J. Kong, H. Li, and C. He, "Flexible, durable, green thermoelectric composite fabrics for textile-based wearable energy harvesting and self-powered sensing," *Composites Science and Technology,* vol. 243, p. 110245, 2023, doi: https://doi.org/10.1016/j.compscitech.2023.110245.

[6] X. He *et al.*, "Layer-by-layer self-assembly of durable, breathable and enhanced performance thermoelectric fabrics for collaborative monitoring of human signal," *Chemical Engineering Journal,* vol. 490, p. 151470, 2024, doi: https://doi.org/10.1016/j.cej.2024.151470.

[7] M. Li *et al.*, "Large-area, wearable, self-powered pressure–temperature sensor based on 3D thermoelectric spacer fabric," *ACS sensors,* vol. 5, no. 8, pp. 2545-2554, 2020, doi: https://doi.org/10.1021/acssensors.0c00870.

[8] X. He, Y. Hao, M. He, X. Qin, L. Wang, and J. Yu, "Stretchable thermoelectric-based self-powered dual-parameter sensors with decoupled temperature and strain sensing," *ACS Applied Materials & Interfaces,* vol. 13, no. 50, pp. 60498-60507, 2021, doi: https://doi.org/10.1021/acsami.1c20456.

[9] Y. Yu, W. Zhu, J. Zhou, Z. Guo, Y. Liu, and Y. Deng, "Wearable respiration sensor for continuous healthcare monitoring using a micro‐thermoelectric generator with rapid response time and chip‐level design," *Advanced Materials Technologies,* vol. 7, no. 8, p. 2101416, 2022, doi: https://doi.org/10.1002/admt.202101416.

[10] C. Zhang *et al.*, "MXene-based wearable thermoelectric respiration sensor," *Nano Energy,* vol. 118, p. 109037, 2023, doi: https://doi.org/10.1016/j.nanoen.2023.109037.

[11] X. He *et al.*, "Continuous manufacture of stretchable and integratable thermoelectric nanofiber yarn for human body energy harvesting and self-powered motion detection," *Chemical Engineering Journal,* vol. 450, p. 137937, 2022, doi: https://doi.org/10.1016/j.cej.2022.137937.

[12] X.-Z. Gao *et al.*, "Self-powered resilient porous sensors with thermoelectric poly (3, 4-ethylenedioxythiophene): poly (styrenesulfonate) and carbon nanotubes for sensitive temperature and pressure dual-mode sensing," *ACS Applied Materials & Interfaces,* vol. 14, no. 38, pp. 43783-43791, 2022, doi: https://doi.org/10.1021/acsami.2c12892.

[13] T. Dinh *et al.*, "Solvent-free fabrication of biodegradable hot-film flow sensor for noninvasive respiratory monitoring," *Journal of Physics D: Applied Physics,* vol. 50, no. 21, p. 215401, 2017, doi: 10.1088/1361-6463/aa6cd6.

[14] H. Wang *et al.*, "Bioinspired fluffy fabric with in situ grown carbon nanotubes for ultrasensitive wearable airflow sensor," *Advanced Materials,* vol. 32, no. 11, p. 1908214, 2020, doi: https://doi.org/10.1002/adma.201908214.

[15] W. Zhou *et al.*, "Bionic adaptive thin‐membranes sensory system based on microspring effect for high‐sensitive airflow perception and noncontact manipulation," *Advanced Functional Materials,* vol. 31, no. 42, p. 2105323, 2021, doi: https://doi.org/10.1002/adfm.202105323.

[16] M. I. Lei and M. Mehregany, "Characterization of thermoelectric properties of heavily doped n-Type polycrystalline silicon carbide thin films," *IEEE transactions on electron devices,* vol. 60, no. 1, pp. 513-517, 2012, doi: https://doi.org/10.1109/TED.2012.2228867.

[17] H. Kitagawa, N. Kado, and Y. Noda, "Preparation of N-type silicon carbide-based thermoelectric materials by spark plasma sintering," *Materials Transactions,* vol. 43, no. 12, pp. 3239-3241, 2002, doi: https://doi.org/10.2320/matertrans.43.3239.

[18] L. Valentín *et al.*, "A comprehensive study of thermoelectric and transport properties of β-silicon carbide nanowires," *Journal of Applied Physics,* vol. 114, no. 18, 2013, doi: https://doi.org/10.1063/1.4829924.

[19] S. Fukuda, T. Kato, Y. Okamoto, H. Nakatsugawa, H. Kitagawa, and S. Yamaguchi, "Thermoelectric properties of single-crystalline SiC and dense sintered SiC for self-cooling devices," *Japanese journal of applied physics,* vol. 50, no. 3R, p. 031301, 2011, doi: 10.1143/JJAP.50.031301.
